# Transcriptional repression of estrogen receptor alpha by YAP reveals the Hippo pathway as therapeutic target for ER^+^ breast cancer

**DOI:** 10.1038/s41467-022-28691-0

**Published:** 2022-02-25

**Authors:** Shenghong Ma, Tracy Tang, Gary Probst, Andrei Konradi, Chunyu Jin, Fulong Li, J. Silvio Gutkind, Xiang-Dong Fu, Kun-Liang Guan

**Affiliations:** 1 Department of Pharmacology and Moores Cancer Center, University of California San Diego, La Jolla, CA 92093 USA; 2 Vivace Therapeutics, San Mateo, CA 94403 USA; 3 Howard Hughes Medical Institute, Department of Medicine, University of California San Diego, La Jolla, CA 92093 USA; 4 Department of Cellular and Molecular Medicine, University of California San Diego, La Jolla, CA 92093 USA

**Keywords:** Breast cancer, HIPPO signalling, High-throughput screening, Breast cancer

## Abstract

Extensive knowledge has been gained on the transcription network controlled by ERα, however, the mechanism underlying *ESR1* (encoding ERα) expression is less understood. We recently discovered that the Hippo pathway is required for the proper expression of *ESR1*. YAP/TAZ are transcription coactivators that are phosphorylated and inhibited by the Hippo pathway kinase LATS. Here we delineated the molecular mechanisms underlying *ESR1* transcription repression by the Hippo pathway. Mechanistically, YAP binds to TEAD to increase local chromatin accessibility to stimulate transcription of nearby genes. Among the YAP target genes, Vestigial-Like Protein 3 (VGLL3) competes with YAP/TAZ for binding to TEAD transcription factor and recruits the NCOR2/SMRT repressor to the super-enhancer of *ESR1* gene, leading to epigenetic alteration and transcriptional silencing. We developed a potent LATS inhibitor VT02956. Targeting the Hippo pathway by VT02956 represses *ESR1* expression and inhibits the growth of ER^+^ breast cancer cells as well as patient-derived tumour organoids. Moreover, histone deacetylase inhibitors, such as Entinostat, induce VGLL3 expression to inhibit ER^+^ breast cancer cells. Our study suggests LATS as unexpected cancer therapeutic targets, especially for endocrine-resistant breast cancers.

## Abstract (web summary)

Hippo signalling is reported to be required for proper ESR1 expression. Here the authors reveal that the transcriptional repression of ESR1 is via LATS-YAP-TEAD-VGLL3 axis and the epigenetic regulation of ESR1 super enhancer in ER + breast cancer.

## Introduction

Breast cancer (BC) is the most common malignancy in women and accounts for a quarter of incidence and more than 15% of female mortality among all cancer types worldwide^1^. Decades of research into the molecular hallmarks classifies breast cancer into four major categories based on the hormone receptors ER/PR and HER2 status with distinct dependencies and clinical outcomes^2^. ER positive breast cancer accounts for 70% and is usually associated with a better prognosis initially, largely due to the persistent functional dependence on ERα activity. Inhibition of ER function by reducing oestrogen levels or administration of selective oestrogen receptor modulators or degraders are the mainstay treatments for ER^+^ breast cancer. However, resistance to endocrine treatment remains a major clinical problem^3^. Among them, recurrent gain-of-function mutations, mainly within the oestrogen binding domain of ERα with reduced ligand dependency, are reported in ~30–40% of metastatic endocrine-resistant ER^+^ breast patients treated with aromatase inhibitors (AIs)^4^. Thus, drugs targeting advanced breast cancers associated with mutant ERα represent a major unmet medical need.

The oestrogen receptor ERα is expressed in multiple tissues and plays fundamental roles in development, morphogenesis, physiological response and malignancy^5^. Over the past decades, extensive knowledge has been gained regarding mechanisms of ERα in transcriptional regulation of downstream target genes. In contrast, the transcriptional regulation of *ESR1* gene itself is incompletely understood^6^. A recent study reported an ER^+^ breast cancer-associated super enhancer located upstream of the *ESR1* transcriptional start site (TSS)^7^. However, the functional connection between the super enhancer and transcriptional regulation of *ESR1* gene is still lacking.

The Hippo pathway is evolutionary conserved from *Drosophila* to mammals, and plays a key role in regulating cell growth and fate decision, organ size and tissue homeostasis^8^. The Hippo pathway integrates a wide range of signals, including cell–cell contact, epithelial polarity, mechanical force, energy status, cellular stress and hormonal factors^9–14^. It consists of the MST/LATS kinase module and the YAP/TAZ-TEAD transcription module. The Hippo pathway core kinases LATS1 and LATS2 (thereafter LATS1/2) inhibits the transcription co-activators YAP/TAZ by phosphorylation, which causes YAP/TAZ sequestration in the cytoplasm and degradation^13,15,16^. While unphosphorylated, YAP/TAZ translocate to the nucleus and bind to TEAD family transcription factors to induce gene^17–19^.

Dysregulation of the Hippo pathway is frequently observed in human cancer. The Hippo pathway kinases, such as LATS, are generally considered as tumour suppressors while YAP/TAZ as oncoproteins^20^. However, recent studies also indicate tumour suppressive activity of YAP/TAZ in metastatic colorectal cancer, haematological cancers and several solid cancers with neural/neuroendocrine origin^21–23^, suggesting cancer type-dependent function of the Hippo pathway in tumorigenesis, but the mechanism has remained elusive. We recently reported that the Hippo signalling is required for the maintenance of *ESR1* gene expression and *LATS* deletion inhibits ER^+^ breast cancer cell growth^24^. Among the different subtypes of breast cancers, TAZ was reported to be highly expressed and function as an oncogene in ER, PR and HER2 receptor triple-negative breast cancers (TNBC)^20^. However, hyperactivation of YAP in mammary epithelia did not induce hyperplasia^25^. Instead, loss of heterozygosity (LOH) of YAP gene locus was observed within breast cancers, although the association with ER status is unclear^26^.

In this study, we delineated the mechanism of *ESR1* transcriptional repression by the LATS-YAP-TEAD-VGLL3 axis. We developed LATS inhibitors that effectively suppress the expression of *ESR1* and revealed a proof of concept that LATS is potential therapeutic target for ER^+^ breast cancer, particularly those with hormone therapy resistant *ESR1* mutations.

## Results

### YAP-TEAD binding increases local chromatin accessibility and target gene expression

**Fig. 1:**
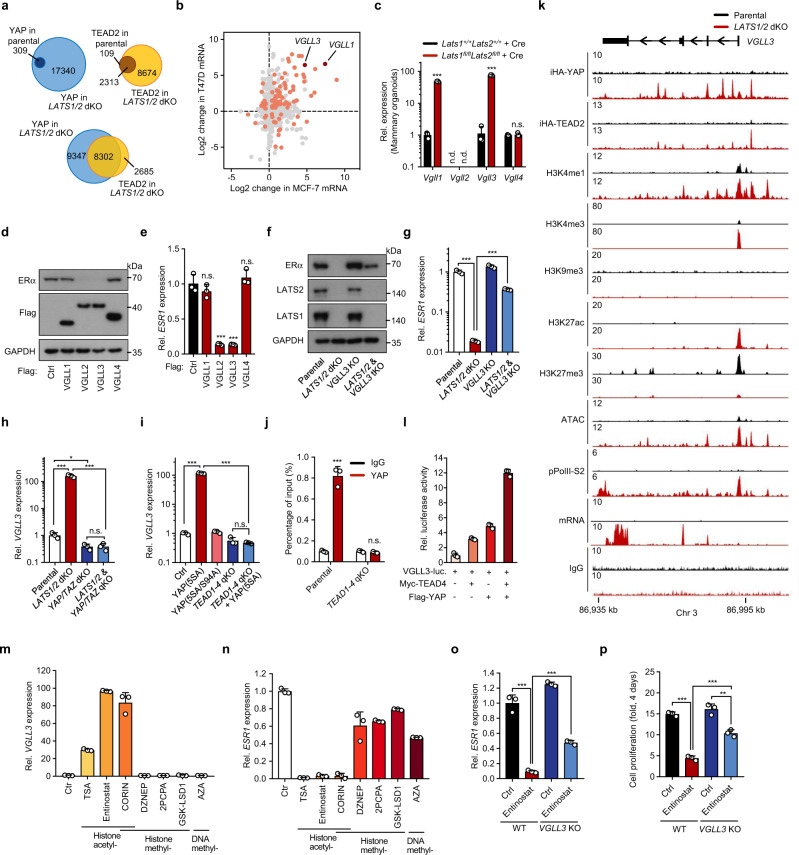
VGLL3 is essential for *ESR1* regulation by the Hippo pathway. **a** Venn diagram showing the overlap of YAP and TEAD2 peaks between parental and *LATS1/2* dKO cells. **b** Differentially expressed genes (DEGs) between *LATS1/2* dKO and WT parental cells with statistical significance (orange/red dots, *p* < 0.01). Only genes that have positive YAP-TEAD ChIP peaks were included in the analyses. **c** qPCR analysis of *VGLL* family genes in organoids derived from the mammary epithelial tissue of *Lats1*^+/+^*Lats2*^+/+^ mice and *Lats1*^fl/fl^*Lats2*^fl/fl^ mice, which both infected with Cre-encoding adenovirus. **d**, **e** MCF-7 cells ectopically expressing VGLLs were subjected to immunoblot with indicated antibodies (**d**) or qRT-PCR for *ESR1* mRNA (**e**). **f**, **g** MCF-7 cells with *LATS1/2* dKO, *VGLL3* KO, *LATS1/LATS2&VGLL3* triple knockout (tKO), or wild-type (Parental) were subjected to immunoblot with indicated antibodies (**f**) or qPCR for *ESR1* (**g**), *n* = 3. **h** MCF-7 cells with *LATS1/2* dKO*, YAP/TAZ* dKO, *LATS1/2* and *YAP/TAZ* quadruple knockout (qKO), or wild-type (Parental) were subjected to qPCR for *VGLL3* mRNA. **i** Wild-type or *TEAD1-4* qKO MCF-7 cells expressing a vector, Flag-YAP(5SA) or Flag-YAP(5SA/S94A) were subjected to qPCR for *VGLL3*. **j** ChIP-qPCR analysis of YAP binding in the *VGLL3* promoter locus between wild-type and *TEAD1-4* qKO cells. **k** Genome track visualisation of indicated signals at the *VGLL3* locus between *LATS1/2* dKO (red) and parental (black) MCF-7 cells. **l** Activation of *VGLL3* luciferase reporter by TEAD4 and YAP. **m**, **n** Total RNA extracted from MCF-7 cells treated by the indicated chemicals for 24 h were subjected to qPCR analysis for *VGLL3* (**n**) or *ESR1* (**m**) mRNA. **o** Wild-type and *VGLL3* KO MCF-7 cells treated with 1 μM Entinostat or DMSO (Ctrl) for 24 h and *ESR1* expression was measured by qPCR. **p** Growth of wild-type and *VGLL3* KO MCF-7 cells in the presence of 1 μM Entinostat or DMSO (Ctrl) for 4 days was determined by cell counting. For **c**, **e**, **h**–**p**, *n* = 3 with mean ±  SEM. Two-sided, unpaired *t*-test for **c**, **e**, **j**; one-way ANOVA Tukey test for **g**–**I**, **o**–**p**. n.d. not detectable, n.s. not significant; **p < 0.01, ***p < 0.001; Source data are provided in the Source Data file.

To understand the mechanism of LATS in *ESR1* regulation, we queried the functional status of ERα in *LATS1/2* deficient cells by performing chromatin immunoprecipitation coupled with next-generation sequencing (ChIP-seq). In accordance, ERα genomic binding was stimulated by 17β-estradiol (E_2_) treatment in parental MCF-7 cells (peaks = 6 and 2854 for −E_2_ and +E_2_, respectively), but this ERα genomic binding was abolished in *LATS1/2* double knock-out (dKO) cells (Supplementary Fig. 1a, b), consistent with our previous report that ESR1 expression was strongly reduced by LATS knockout^24^. In order to delineate the Hippo-YAP-*ESR1* axis, we characterised the cistrome for the transcriptional co-factor YAP and its binding transcription factor TEAD. We titrated the concentrations of doxycycline to achieve inducible expression of iHA-YAP and iHA-TEAD2 similar to their endogenous levels (Supplementary Fig. 1c, d). As expected, inactivation of Hippo signalling by *LATS1/2* dKO markedly increased the number of iHA-YAP binding peaks (Fig. 1a and Supplementary Fig. 1e, f). Interestingly, the iHA-TEAD2 binding sites were also increased in *LATS1/2* dKO cells, albeit to a lower extent compared with YAP (Fig. 1a and Supplementary Fig. 1e, f). TEAD recognition motifs were the top enriched motifs in both iHA-YAP and iHA-TEAD2 binding peaks (Supplementary Fig. 1g, h). The next enriched motifs were binding sites for the JUN-FOS family of transcriptional factors, consistent with the findings in previous studies that YAP-TEAD co-enriched with JUN-FOS binding sites^19,27^. In all, 79% (8674/10987) of all TEAD2 binding peaks directly overlapped with 47% (8302/17649) of YAP peaks (Fig. 1a). The higher number of YAP peaks compared to TEAD2 could possibly been caused by additional DNA binding partners for YAP, potential competition between iHA-TEAD2 and endogenous TEAD protein, or different ChIP efficiency between iHA-YAP and iAH-TEAD. In addition, a recent YAP/TEAD chromatin profiling performed in MCF-7 cells also reported larger number of YAP peaks compared to TEAD^28^. Together, these observations demonstrate that TEAD is the primary YAP target transcription factor in MCF7, and LATS deficiency not only increases the genomic binding of YAP, but also TEAD.

Next, we set out to determine the functional implication of YAP and TEAD genomic binding. Out of the 8302 YAP-TEAD2 co-binding events, several active histone marks were highly enriched, especially acetylation of histone 3 lysine 27 (H3K27ac), mono-methylation of histone 3 lysine 4 of (H3K4me1) and di-methylation of histone 3 lysine 4 (H3K4me2) (Supplementary Fig. 1i). In contrast, repressive histone marks were not enriched. These data indicate that YAP-TEAD binding is positively associated with histone modifications linked to gene activation. We further tested whether YAP-TEAD binding could actively remodel local chromatin to a pro-active status. We performed Assay for Transposase-Accessible Chromatin coupled with high-throughput sequencing (ATAC-seq) for chromatin accessibility^29^. *LATS1/2* deficiency did not result in a global upregulation of ATAC signals (Supplementary Fig. 2a, d, g). However, *LATS1/2* dKO increased chromatin accessibility in the YAP-TEAD binding peaks (Supplementary Fig. 2b, e, g). While the chromatin accessibility in the ERα binding peaks was reduced in *LATS1/2* dKO cells (Supplementary Fig. 2c, f, g).

By interrogating the correlation between YAP-TEAD or ERα binding peaks to the closest transcriptional start sites (TSS), we observed that the probability of transcriptional activation of genes next to YAP-TEAD binding peaks was significantly elevated in the *LATS1/2* dKO cells (Supplementary Fig. 2h). Conversely, the expression of genes next to ERα binding sites was suppressed in the *LATS1/2* dKO cells (Supplementary Fig. 2h). Taken together, our data support a model that YAP-TEAD binding increases local chromatin accessibility to stimulate transcription of nearby genes.

As the YAP protein does not harbour chromatin remodelling domains, we hypothesised that YAP recruits chromatin modifiers to alter local chromatin status. We used TurboID technology^30^ to search for YAP interacting proteins. Besides the known binding partners of TEAD and LATS, multiple components of the SWI/SNF complex and MED23, a component of the mediator complex, were also detected as YAP interacting proteins (Supplementary Fig. 2i). The SWI/SNF complex has broad roles in chromatin remodelling and transcriptional regulation^31^. Thus, our results suggest that YAP-TEAD stimulates downstream target gene transcription by increasing the local chromatin accessibility, possibly through the recruitment of chromatin remodelling complexes.

### VGLL3 mediates Hippo signalling to inhibit *ESR1* expression

Since YAP is associated with gene activation, we speculated that *ESR1* transcription is indirectly inhibited by active YAP in *LATS1/2* dKO cells. We further posit that YAP target gene(s) may be responsible for *ESR1* transcriptional repression. Given the conserved nature of the Hippo-YAP-*ESR1* axis in different cell lines and biological systems, we compared the differentially expressed genes (DEG), which also have YAP-TEAD binding sites, between *LATS1/2* dKO and the parental cells of MCF-7 and T47D (Fig. 1b). Among the top commonly YAP-TEAD target DEGs, the VGLL family genes *VGLL1* and *VGLL3* were particularly noteworthy (Fig. 1b and Supplementary Fig. 3a) because VGLL4, a VGLL family protein, has been reported to inhibit YAP-TEAD transcriptional activity by displacing YAP from TEAD^32,33^. The upregulation of *VGLL* family genes by *LATS1/2* deletion were further confirmed in ER^+^ breast cancer cell lines, as well as in ER^+^ tissue organoids, including mammary epithelial organoids, endometrial organoids and fallopian tube organoids (Fig. 1c and Supplementary Fig. 3b–d). Notably, only *VGLL3* was commonly upregulated by *LATS1/2* deletion in all cells and tissues tested. In addition, knockout of the Hippo upstream component NF2, which is required to maintain LATS activity and *ESR1* expression, also induced *VGLL1* and *VGLL3* (Supplementary Fig. 3e).

As the sequence similarity among the VGLL family proteins were low and mainly limited to the small Vg domain (Supplementary Fig. 3a), we tested whether VGLL family protein(s) could differentially inhibit *ESR1* expression. Ectopic expression of VGLL2 or VGLL3, but not VGLL1 nor VGLL4, reduced both mRNA and protein levels of *ESR1* (Fig. 1d, e and Supplementary Fig. 3f–h). This is in line with the fact that VGLL2 and VGLL3 share the highest sequence homology (Supplementary Fig. 3a). Because *VGLL2* was not induced by *LATS1/2* dKO in breast cells and was mainly expressed in muscle cells (Supplementary Fig. 3i), we therefore focused on VGLL3. Deletion of endogenous *VGLL3* had little effect on the basal *ESR1* expression, but strongly diminished, although not completely, the *LATS1/2* dKO-triggered *ESR1* downregulation (Fig. 1f, g and Supplementary Fig. 3j). Together, these observations support a model that VGLL3 play a key role in mediating Hippo signalling to repress *ESR1* expression.

We next asked how *VGLL3* is regulated by the Hippo pathway. The upregulation of *VGLL3* transcript in *LATS1/2* dKO cells was reversed by putting-back wild-type but not kinase inactive Lats2 mutant (KR) (Supplementary Fig. 3k). Expression of an active YAP(5SA), which has all five inhibitory LATS phosphorylation serine residues replaced by alanines, robustly induced *VGLL3* expression (Supplementary Fig. 3l). Conversely, YAP/TAZ dKO blocked *VGLL3* induction by *LATS1/2* dKO (Fig. 1h). Moreover, the TEAD binding deficient YAP(5SA/S94A), which has the TEAD binding essential serine 94 replaced by an alanine, failed to induce VGLL3 (Supplementary Fig. 3l). Consistently, depletion of all four *TEAD1-4* abolished *VGLL3* induction by the active YAP (Fig. 1i). YAP binding on the VGLL3 promoter region was also abolished in *TEAD1-4* quadruple knock out (qKO) cells (Fig. 1j). These data establish a LATS-YAP-TEAD-VGLL3-*ESR1* axis.

To gain insights into *VGLL3* induction by YAP-TEAD, we characterised chromatin status of the *VGLL3* genomic locus by performing extensive chromatin profilings: ChIP-seq for YAP and TEAD; Cleavage Under Targets and Tagmentation with high-throughput sequencing (CUT&Tag-seq) for histone marks H3K4me1, H3K4me3, H3K9me3, H3K27ac and H3K27me3; and ATAC-seq (Fig. 1k). We observed that *LATS1/2* dKO increased the binding of YAP and TEAD at multiple sites within the *VGLL3* genomic locus, supporting the notion of *VGLL3* as a direct target gene of YAP-TEAD. *LATS1/2* dKO also increased active chromatin marks, including H3K4me1, H3K4me3 and H3K27ac, as well as decreased abundance of repressive mark H3K27me3. As expected, a strong induction of ATAC signals along with higher RNA polymerase II transcriptional activity were observed at *VGLL3* locus in *LATS1/2* dKO cells (Fig. 1k). These results support that YAP-TEAD stimulates the *VGLL3* expression by inducing local chromatin remodelling.

Next, we constructed a luciferase reporter driven by the *VGLL3* promoter, which contains the YAP-TEAD binding region. Expression of YAP and TEAD4 synergistically increased the *VGLL3* reporter activity (Fig. 1l), reinforcing a direct role of YAP-TEAD in VGLL3 induction. We further analysed the gene expression data from several public breast cancer datasets. As expected, there is a strong positive correlation between *CTGF* and *CYR61*, two well-known YAP/TAZ target genes (Supplementary Fig. 3m–p). Interestingly, we also observed a strong positive correlation between *VGLL3* expression with either *CTGF* or *CYR61* mRNA in multiple cancer types, including breast cancer (Supplementary Fig. 3m–p). In addition, we further compared the expression levels of LATS1/2 and YAP/TEAD with VGLL3 as well as known YAP target genes *CTGF* and *CYR61*. We observed a strong positive correlation between *LATS2*, YAP (encoded by *YAP1* gene), TAZ (encoded by *WWTR1*) and these YAP/TEAD direct target genes (Supplementary Fig. 3q). LATS2 kinase is not only a negative regulator of YAP, but also a direct YAP/TEAD target gene in a negative-feedback loop^34,35^. Collectively, these data provide in vivo evidence supporting *VGLL3* as a YAP-TEAD target gene in breast cancers.

### VGLL3 mediates the anti-tumour activity of histone deacetylase inhibitors in ER^+^ breast cancer

Considering that the elevated transcription of *VGLL3* by YAP-TEAD is associated with local chromatin remodelling, including the induction of several active histone marks, we performed a small-scale screening with multiple small molecules targeting different epigenetic modifiers. Interestingly, the histone deacetylase inhibitors TSA, Entinostat and CORIN robustly increased *VGLL3* expression whereas molecules targeting histone methylation or DNA methylation had little effect (Fig. 1m). In accordance with the *VGLL3* induction, TSA, Entinostat and CORIN strongly repressed *ESR1* expression (Fig. 1n).

Entinostat, a benzamide class I specific HDAC inhibitor, is in clinical trials for multiple cancer types, including ER^+^ breast cancers^36^. We observed that the Entinostat-induced *ESR1* downregulation was diminished in *VGLL3* KO MCF-7 cells (Fig. 1o). Moreover, Entinostat-induced inhibition of cell growth was significantly blocked by *VGLL3* KO (Fig. 1p). These observations indicate that *VGLL3* induction contributes to the *ESR1* repression and anti-tumour effect of Entinostat, thus revealing a potential mechanism of action for histone deacetylase inhibitor drugs in ER^+^ breast cancers.

### VGLL3 inhibits ERα expression by recruiting NCOR2 repressor

**Fig. 2:**
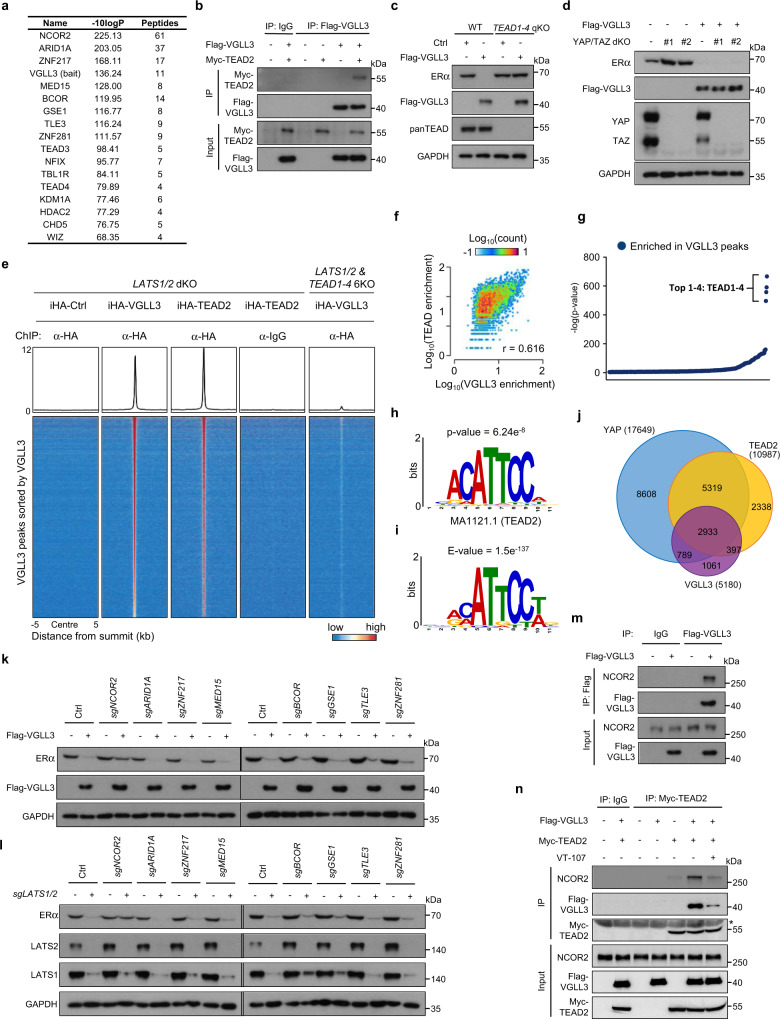
VGLL3 represses *ESR1* transcription by binding TEAD and recruiting NCOR2. **a** List of transcriptional regulators identified by VGLL3-TurboID mass spectrometry. **b** VGLL3 interacts with TEAD. MCF-7 cells were transiently transfected with plasmids expressing the indicated proteins. Protein–protein interaction was examined by IP-western blot using the indicated antibodies. **c** TEAD is required for VGLL3-induced ERα downregulation. Wild-type and *TEAD1-4* qKO MCF-7 cells expressing a control vector or Flag-*VGLL3* cDNA were subjected to immunoblot analysis. **d** YAP/TAZ are dispensable for VGLL3-induced ERα downregulation. Wild-type or YAP/TAZ dKO MCF-7 cells transduced with control vector or Flag-*VGLL3* cDNA were subjected to immunoblot analysis. **e** Heatmap and line graph of the ChIP-seq profiles for doxycycline (dox) inducible HA-tagged VGLL3 (iHA-VGLL3), TEAD2 (iHA-TEAD2), and control vector (iHA-Ctrl) in *LATS1/2* deficient cells or iHA-VGLL3 in *LATS1/2* & *TEAD1-4* 6KO cells at the summits of iHA-VGLL3 peaks. **f** Scatterplots depicting the correlation between iHA-VGLL3 and iHA-TEAD2 ChIP-seq signals in *LATS1/2* deficient cells. **g** Enrichment of the TEAD motif in VGLL3 ChIP-seq. MEME-AME Motif enrichment analysis for VGLL3 binding peaks against JASPAR CORE database (1404 profiles) and ordered by log (*p*-value). **h** The TEAD motif (JASPAR Matrix ID: MA1121.1) was enriched at VGLL3 ChIP-seq peaks. **i** De novo motif analysis for the VGLL3 binding peaks by MEME-Suit. **j** Venn diagram showing the overlap of VGLL3 (purple), TEAD2 (yellow) or YAP (blue) peaks in *LATS1/2* dKO MCF-7 cells. **k** NCOR2 is required for VGLL3 to repress *ESR1*. MCF-7 cells with CRISPR-cas9 sgRNA targeting individual putative VGLL3 binding partners were transfected with Flag-VGLL3 or control vector. Immunoblot analysis was performed with the indicated antibodies. **l** NCOR2 is required for *ESR1* repression by *LATS1/2* deletion. MCF-7 cells were infected with CRISPR sgRNA targeting individual VGLL3 binding partners or in combination with sgRNA targeting *LATS1/2*. **m** VGLL3 interacts with NCOR2. MCF-7 cells expressing Flag-VGLL3, or control vector, were immunoprecipitated with Flag antibody. Western blotting for co-precipitated endogenous NCOR2 was determined. **n** VGLL3 mediates TEAD-VGLL3-NCOR2 complex formation. MCF7 transfected with indicated proteins and immunoprecipitated with anti-Myc-tag or IgG control antibodies were subjected to immunoblot with indicated antibodies. Asterisk indicates non-specific band. Source data are provided in the Source Data file.

To gain mechanistic insight into the *ESR1* repression by VGLL3, we searched for VGLL3 interacting proteins by performing VGLL3 TurboID coupled with mass spectrometry. Among the top candidates related to transcriptional regulation were TEAD family proteins (Fig. 2a). This is not unexpected considering the reported interaction between VGLL4 and TEAD^32,33^. We thus validated the interaction between TEAD and VGLL3 by reciprocal co-immunoprecipitation and western blotting (Fig. 2b and Supplementary Fig. 4a). Deletion experiments showed that the Vg domain, which is 24 residues in length and the only conserved region among the VGLL1-4 family proteins^37^, was essential for TEAD binding (Supplementary Fig. 4b, c). VGLL3-AA, which has mutations of two conserved residues within the Vg domain, disrupted the interaction with TEAD and was inactive to supress *ESR1* expression (Supplementary Fig. 4d, e), indicating a possible role of TEAD in *ESR1* repression by VGLL3. TEAD1-4 proteins are composed of two major domains, the N-terminal DNA binding domain (TEA) and the C-terminal YAP binding domain (YBD) (Supplementary Fig. 4f). We found that the TEAD YBD domain was essential for VGLL3 binding (Supplementary Fig. 4g). As YAP and VGLL3 both bind to the YBD domain of TEAD, we asked whether VGLL3 could compete with YAP for TEAD binding. Indeed, VGLL3, but not the VGLL3-AA mutant, competed with YAP for TEAD binding while VGLL3 did not interact with YAP (Supplementary Fig. 4h–j). These data indicates that TEAD can form two mutually exclusive complexes, with either YAP to activate transcription or VGLL3 to repress transcription.

We asked the functional relevance of TEAD in VGLL3-dependent *ESR1* repression. The reduction of *ESR1* expression by VGLL3 was completely blocked by depletion of *TEAD1-4* (Fig. 2c). In addition, the TEAD inhibitor VT-107^38^ ameliorated the ERα downregulation caused by *LATS1/2* dKO (Supplementary Fig. 4k). In contrast, YAP/TAZ was not required for VGLL3-induced *ESR1* repression (Fig. 2d). We also asked whether YAP could directly regulate *ESR1* expression. We thus ectopically expressed the constitutively active YAP(5SA) in parental and *VGLL3* knockout (KO) MCF7 cells. We observed that *VGLL3* KO largely blocked YAP(5SA)-induced *ESR1* reduction (Supplementary Fig. 4l). Since VGLL3 is a YAP target gene, we posit that YAP indirectly represses *ESR1* through the induction of VGLL3. These data show that VGLL3 represses *ESR1* expression by binding to TEAD without direct involvement of YAP/TAZ, whereas YAP/TAZ stimulate VGLL3 expression.

Next, we performed cistrome profiling for VGLL3 with Tet-On inducible system (iHA-VGLL3) and compared that with the binding landscape of TEAD. The interplay between VGLL3 and TEAD was evidenced by the following observations. Firstly, the VGLL3 chromatin binding was co-enriched, and the intensity was positively correlated with TEAD peaks (Fig. 2e, f). Secondary, the TEAD motifs were the most significantly enriched motifs in the VGLL3 peaks among 579 JASPAR CORE vertebrate transcriptional factor dataset (Fig. 2g, h). Thirdly, de novo motif analysis also generated a motif resembles the TEAD recognition motif (Fig. 2h, i). Lastly, deletion of *TEAD1-4* genes abolished VGLL3 genomic binding (Fig. 2e). The above results demonstrate that TEAD mediates VGLL3 chromatin binding.

Since TEADs are binding partners for both VGLL3 and YAP, we asked whether VGLL3 or YAP might have preference for different TEAD peaks. We thus performed Venn diagram comparison between VGLL3, TEAD and YAP ChIP binding peaks obtained from the *LATS1/2* dKO cells. We observed a high percentage of overlap between VGLL3 and TEAD (Fig. 2j). In addition, YAP also co-localised with the VGLL3^+^/TEAD^+^ peaks, suggesting that YAP and VGLL3 proteins dynamically compete for binding to similar TEAD peaks when a population of cells were analysed. Single cell ChIP-Seq experiments might be needed to demonate the mutatual exclusive binding of YAP and VGLL3 to genomic TEAD sites.

Besides TEAD, VGLL3-turboID also identified multiple proteins known to be involved in transcriptional regulation (Fig. 2a). We assessed the functional effect of these putative VGLL3 interacting proteins individually. Depletion of *NCOR2* (a core component of SMRT transcriptional repressor complex), but not other candidates, blocked the *ESR1* downregulation caused by ectopically expressed Flag-VGLL3 (Fig. 2k). Knockout of NCOR2 also ablated the *LATS1/2* dKO-induced *ESR1* downregulation (Fig. 2l). We confirmed the interaction between NCOR2 and VGLL3 by co-immunoprecipitation (Fig. 2m). In addition, shRNA mediated *NCOR2* knock-down by two different guide RNAs efficiently blocked the downregulation of *ESR1* caused by either *LATS1/2* dKO or ectopic VGLL3 expression (Supplementary Fig. 4m, n).

Next, we asked whether VGLL3 might tether the TEAD and NCOR2 interaction. We conducted TEAD2 Co-IP with NCOR2 in the absence or presence of VGLL3, or TEAD inhibitor VT-107^38^. We found that VGLL3 increased the Co-IP efficiency between TEAD2 and NCOR2 (Fig. 2n), supporting a notion that VGLL3 works as a linker in TEAD-VGLL3-NCOR2 complex. The TEAD inhibitor VT-107 disrupted the interaction of TEAD with either VGLL3 or NCOR2 (Fig. 2n). Considering the similar binding mechanism of VGLL3 or YAP with TEAD, this observation was consistent with previous observations that VT-107 directly binds to TEAD and disrupts YAP/TAZ-TEAD interaction^38^. Collectively, our results demonstrate that NCOR2 recruitment by VGLL3 is critical for *ESR1* downregulation by the Hippo signalling. Furthermore, VGLL3 actively participates in gene repression, as different from the current model of a simple displacement of YAP from TEAD by VGLL4^32,33^.

### The VGLL3-TEAD targets the super enhancer of *ESR1*

**Fig. 3:**
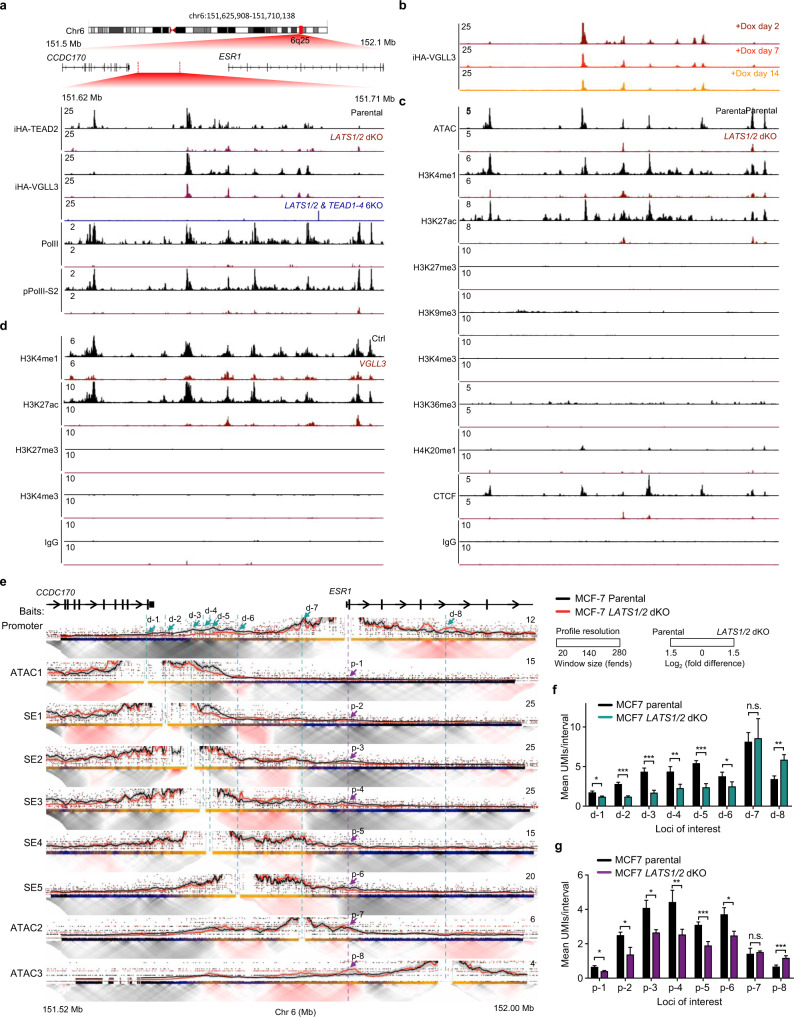
The Hippo-VGLL3 targets the super enhancer locus of *ESR1*. **a** Co-enrichment of TEAD2 and VGLL3 at the *ESR1* super enhancer locus. Genome track visualisation of iHA-TEAD2, iHA-VGLL3, PolII and pPolII-S2 signals at the *ESR1* distal super enhancer locus for *LATS1/2* dKO (red), parental (black) cells, and iHA-VGLL3 in *LATS1/2* & *TEAD1-4* 6KO cells (blue). **b** Genome track comparison of iHA-VGLL3 at the *ESR1* super enhancer locus upon doxycycline (dox) induction of iHA-VGLL3 at day 2, day 7 and day 14. **c** *LATS1/2* deficiency alters chromatin status of the *ESR1* super enhancer locus. Genome track visualisation of ATAC, H3K4me1, H3K27ac, H3K27me3, H3K9me3, H3K4me3, H3K36me3, H4K20me1, CTCF and IgG signals at the *ESR1* distal super enhancer locus between *LATS1/2* deficient (red) and parental (black) MCF-7 cells. **d** VGLL3 expression phenocopies the *ESR1* super enhancer histone modifications associated with *LATS1/2* deletion. Genome track visualisation of H3K4me1, H3K27ac, H3K27me3 and H3K4me3 signals at the *ESR1* distal super enhancer locus by ectopic expression of *VGLL3* (red) or control vector (black) in MCF-7 cells. **e** *LATS1/2* deficiency decreases the interaction between *ESR1* promoter and the distal super enhancer locus. In the virtual in-situ umi-4C plots, the dotted line and arrow denotes the viewpoint drawn from *ESR1* promoter locus (purple) or selected genomic intervals (cyan). Domainogram colour (log_2_ fold difference) are relative to the maximum profile to the presented genomic window. SE1-5 represent TEAD-VGLL3 positive peaks within the *ESR1* super enhancer locus whereas ATAC1-3 are putative distal regulatory elements outside the super enhancer. **f**, **g** *LATS1/2* dKO diminishes the distal interaction between the *ESR1* promoter and the super enhancer locus. Quantification of the UMI-4C contact intensities between *ESR1* promoter locus and eight genomic intervals (ATAC1-3 and SE1-5) in the *ESR1* regulatory region. *ESR1* promoter locus (**f**) or eight genomic intervals (**g**) were used as 4C baits. Error bars, estimated binomial s.d.; n.s. not significant; **p < 0.01, ***p < 0.001; Source data are provided in the Source Data file.

The expression of *ESR1* in ER positive breast cancer cells was reported to be associated with a distal super enhancer^7^, which localised in the same topologically associating domain (TAD) with *ESR1* gene (Supplementary Fig. 5a) and characterised by high H3K27Ac, high H3K4me1, low H3K27me3 and DNase hypersensitivity (Supplementary Fig. 5b). Interestingly, our analysis of the ENCODE dataset revealed that TEAD4 was present within the *ESR1* super-enhancer locus (Supplementary Fig. 5b). Indeed, we observed a strong and concordant binding of both VGLL3 and TEAD in the super enhancer (Fig. 3a). The TEAD family proteins were essential for VGLL3 binding on the super enhancer locus as *TEAD1-4* qKO abolished VGLL3 binding (Fig. 3a). Interestingly, unlike the increased global TEAD binding in *LATS1/2* dKO cells, the binding intensity of both VGLL3 and TEAD2 on the *ESR1* super enhancer was reduced after deletion of *LATS1/2* (Fig. 3a). We further performed time course CUT&Tag-seq for VGLL3 and observed a time-dependent decrease of VGLL3 binding (Fig. 3b). We speculated that *LATS1/2* deficiency remodels the chromatin status of the *ESR1* super enhancer to reduce chromatin accessibility. Indeed, ATAC-seq data confirmed a loss of chromatin openness accompanied with reduction of active enhancer marks H3K4me1 and H3K27ac (Fig. 3c). These data support a notion that *LATS1/2* dKO alters the local chromatin status and shuts down the *ESR1* super enhancer. We further hypothesised that VGLL3 protein might be responsible for this chromatin remodelling at the *ESR1* super enhancer. We conducted CUT&Tag profiling for multiple enhancer marks and found that inducible expression of VGLL3 alone was sufficient to decrease H3K4me1 and H3K27ac, which phenocopies the effects in *LATS1/2* dKO cells (Fig. 3d). These results confirm a key role of VGLL3 in the Hippo-*ESR1* axis regulation.

During chromatin profiling, we noted a significant reduction of CCCTC-binding factor (CTCF) signals at the super enhancer locus in the *LATS1/2* dKO cells (Fig. 3c). CTCF can organise chromosomal architecture and is important for distal DNA-DNA interaction^39^. We examined the effect of Hippo pathway on the distal interaction between the *ESR1* super enhancer and promoter. We modified a targeted chromosome conformation capture (3C) with unique molecular identifiers (UMI-4C-seq)^40^ and in-situ 3C library generation technology^41^ to achieve quantitative and multiplexed interactome with improved sensitivity. We designed several contact profiling baits targeting the *ESR1* promoter locus as well as multiple regions within the super enhancer with co-binding of TEAD and VGLL3 proteins (SE1 to SE5), and additional distal ATAC peaks located both upstream (ATAC1 and ATAC2) and downstream (ATAC3) of the *ESR1* TSS (Fig. 3e). When the *ESR1* promoter was used as the in-situ UMI-4C bait, we observed a significant reduction of the contact strength towards the super enhancer in the *LATS1/2* dKO cells (d-2 to d-6) (Fig. 3e, f). In contrast, the relative contact strength between ESR1 promoter and ATAC2 (d-7) or ATAC3 (d-8), the two ATAC peaks outside the super enhancer region, was either unchanged or increased (Fig. 3e, f). Reciprocally, when the distal loci were used as the baits, the interaction with *ESR1* promoter was reduced for the super enhancer baits (p-2 to p-6) but not the ATAC2 bait (p-7) nor ATAC3 bait (p-8) in the *LATS1/2* dKO cells (Fig. 3g). The reduced interaction between the super enhancer and *ESR1* promoter locus in *LATS1/2* deficient cells was further validated by multiplexed in-situ UMI-4C-seq in another ER^+^ breast cancer line T47D (Supplementary Fig. 5c–e). In addition, either *VGLL3* KO or treatment with the TEAD inhibitor VT-107 could ameliorate the decreased contact between *ESR1* promoter and super enhancer locus caused by *LATS1/2* dKO (Supplementary Fig. 5f). Taken together, these data demonstrate that LATS is required to maintain the interaction between *ESR1* promoter and super enhancer, thus sustaining *ESR1* expression.

### Hippo-ER axis in breast tumours and patient-derived organoids

In an effect to explore the clinical implications of the Hippo-YAP/TAZ pathway in breast cancers, we analysed gene copy number variations and expression levels of the YAP and TAZ in the BRCA cohort from TCGA dataset. *WWTR1* (gene encoding TAZ) was amplified in 24% of ER^-^ BRCA (Supplementary Fig. 6a), which is consistent with previous reports of oncogenic role of TAZ in triple-negative breast cancer^42,43^. Notably, in the ER^+^ cohort of BRCA, *YAP1* (gene encoding YAP) copy number was decreased in 38% of tumours (Supplementary Fig. 6b), suggesting a potential tumour suppressor function of YAP in ER^+^ BRCA. In line with this, expression levels of *YAP1* and *WWTR1* as well as their target genes *CTGF* and *CYR61* were significantly decreased in ER^+^ BRCA relative to their non-tumour tissues (Supplementary Fig. 6c, d).

Next, we conducted the analysis using a recent single cell dataset, which grouped single cells into different cancer subtypes and normal epithelial cell types^44^. We observed decreased expression of *YAP1*, *WWTR1*, *CTGF* and *CYR61* in Cancer Luminal type A/B cells when compared with normal mature luminal cells (Supplementary Fig. 6e). The downregulation of YAP1 was more dramatic in the Cancer Luminal Type B. We also analysed the YAP1 expression level and survival. Low expression of *YAP1* was associated with reduced survival in ER^+^, but not in ER^-^, breast cancer patients (Supplementary Fig. 6f, g). We next explored the clinical relevance of VGLL3 in ER^+^ breast cancer. *VGLL3* was significantly downregulated in Luminal Type B breast cancers (Supplementary Fig. 6h, i). Among the ER + Luminal type A/B breast cancers, *VGLL3* expression negatively correlated with ERα status (Supplementary Fig. 6j). Moreover, low *VGLL3* expression was associated with reduced survival in ER^+^ but not in other types of breast cancers (Supplementary Fig. 6k–m). In accordance, we observed that ectopic expression of VGLL3 inhibited MCF-7 cell growth (Supplementary Fig. 6n), consistent with a role of VGLL3 in repressing *ESR1* expression.

**Fig. 4:**
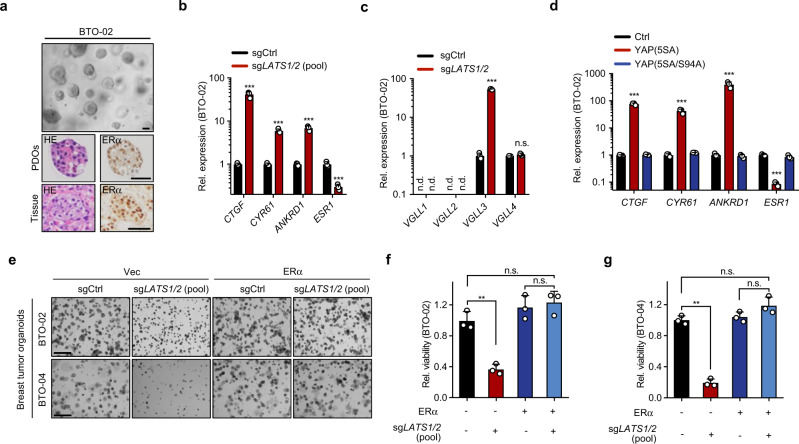
LATS is important to maintain *ESR1* expression and growth of patient-derived breast tumour organoids. **a** Establishment of ER^+^ breast tumour organoids. Representative bright field image, H&E staining and ERα immunohistochemistry of tumour organoid and the matching patient biopsy tissue. Scale bar, 50 μm. **b**, **c** *LATS1/2* deletion induces YAP target genes and decreases *ESR1*. *LATS1/2* were deleted by lentivirus mediated CRISPR in tumour organoid (BTO-02). Expression of YAP-TEAD target genes, *ESR1* (**b**), and *VGLL* family genes (**c**) were determined by qPCR. **d** YAP inhibits *ESR1* expression in patient-derived breast tumour organoids. qPCR analysis of YAP target genes and *ESR1* in BTO-02 transduced with control vector, constitutively active YAP(5SA), or the TEAD binding defective YAP(5SA/S94A). **e**–**g** ERα mediates the growth inhibitory effect of LATS deficiency in ER^+^ breast tumour organoids. Organoids BTO-02 and BTO-04 infected with lentivirus expressing ERα encoding gene or a control vector followed by further transduction of CRISPR-cas9 targeting *LATS1/2* or non-specific sequence were seeded in Matrigel. Representative images of colony growth (**e**), quantification of cell growth of BTO-02 (**f**) and BTO-04 (**g**). Scale bar for **e**, 150 μm. For **b**–**d**, **f**, **g**, *n* = 3. Mean ±  SEM. Two-sided, unpaired *t*-test for **b**–**d**; one-way ANOVA Tukey test for **f**, **g**; n.d. not detectable; n.s. not significant; **p < 0.01, ***p < 0.001; Source data are provided in the Source Data file.

We established ER^+^ breast tumour organoids (BTO) from surgically resected tumours. Morphological similarity and ERα expression between BTOs and the corresponding tumours were confirmed by histological evaluation (Fig. 4a). The established organoids retain ER functionality, including E_2_-stimulated gene expression and response to tamoxifen and fulvestrant treatment (Supplementary Fig. 6o, p). We next examined the Hippo-*ESR1* axis regulation in ER^+^ BTOs by using lentivirus CRISPR-cas9 mediated *LATS1/2* deletion. We observed a strong transcriptional induction of multiple YAP-TEAD target genes, including *CTGF*, *CRY61*, *ANKRD1* and *VGLL3*, upon *LATS1/2* deletion (Fig. 4b, c). Concomitantly, *LATS1/2* dKO diminished the expression of *ESR1* in BTOs (Fig. 4b). In addition, expression of YAP(5SA), but not the TEAD binding deficient YAP(5SA/S94A), increased VGLL3 expression and suppressed *ESR1* expression (Fig. 4d and Supplementary Fig. 6q). Furthermore, ectopic expression of VGLL3 also significantly inhibited *ESR1* expression (Supplementary Fig. 6r). These data demonstrate that the Hippo-YAP-VGLL3-*ESR1* axis is intact in patient-derived BTOs.

Next, we tested the function of the Hippo pathway in BTOs by three-dimensional (3D) growth assay. Deletion of *LATS1/2* significantly reduced cell growth of ER^+^ BTOs (Fig. 4e–g). Importantly, ectopic expression of ERα blunted the inhibitory effects of *LATS1/2* dKO on BTO growth (Fig. 4e–g and Supplementary Fig. 6s). Based on the above data, we conclude that LATS1/2 are required for the growth of ER^+^ BTO by maintaining ERα expression.

### LATS inhibitor VT02956 impedes *ESR1* transcription

**Fig. 5:**
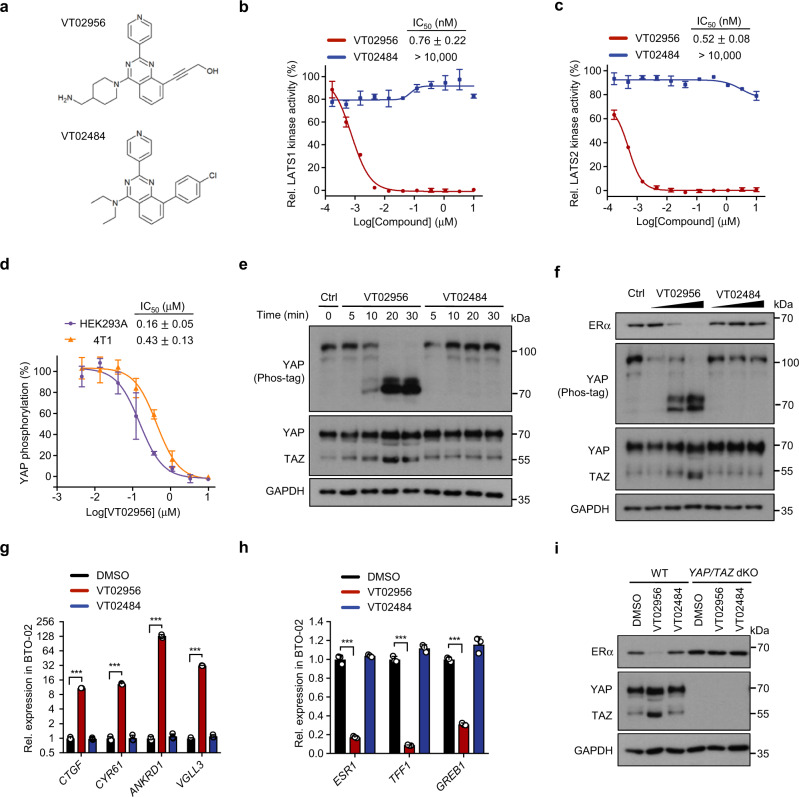
Pharmacological LATS inhibition suppresses *ESR1* transcription. **a** Chemical structure of the LATS inhibitor VT02956 and its inactive analogue VT02484. **b**, **c** Inhibition of LATS kinase activity by VT02956. Activity of the LATS1 (**b**) and LATS2 (**c**) kinases were measured in the presence of increasing concentration of VT02956 or VT02484 in vitro. **d** VT02956 inhibits YAP phosphorylation in cells. YAP phosphorylation was measured in the presence of increasing concentration of VT02956 in HEK293A (purple curve) or 4T1 (orange curve) cells. The HTRF phospho-YAP assay was carried out as described in the Methods. For **b**–**d**, *n* = 2. **e** Time course of VT02956-induced YAP/TAZ dephosphorylation. Immunoblot of the indicated proteins in HEK293A cells treated with VT02956 or VT02484 at 2 μM for the indicated time points. Phot-tag detects reduced YAP mobility due to phosphorylation at multiple sites. **f** VT02956 dose-dependently induces YAP/TAZ dephosphorylation and ERα reduction. MCF-7 cells were treated with increased dose (0.1, 0.5 and 2 μM) of the LATS inhibitor VT02956 or inactive analogue VT02484 for 2 days prior to immunoblot analysis. **g**, **h** LATS inhibitor VT02956 increases the expression of YAP target genes, reduces expression of *ESR1* and its target genes in MCF-7 cells. qPCR analysis of YAP/TAZ target genes (**g**) or *ESR1* and ERα target genes (**h**) in breast tumour organoids BTO-02 treated with 2 μM of VT02956 or VTVT02484 for 2 days as indicated. For **g**, **h**, *n* = 3. **i** YAP/TAZ are required for VT02956-induced ERα downregulation. Wild-type (WT) or *YAP/TAZ* dKO MCF-7 cells were treated with 2 μM VT02956, 2 μM VT02484, or DMSO control for 2 days, and then analysed by immunoblot for ERα expression. Mean ± SEM. Two-sided, unpaired *t*-test for **g**, **h**; ***p < 0.001. Source data are provided in the Source Data file.

To further evaluate the therapeutic value of targeting LATS, we conducted a high throughput screen for LATS kinase inhibitors using an in vitro LATS kinase assay. Out of ~17,000 compounds screened, 230 were found to be positive with IC_50_ < 3 μM (details see Methods section). Further screening and extensive structure-activity relationship study resulted in a potent LATS inhibitor VT02956 (Fig. 5a and Supplementary Fig. 7a). VT02956 inhibited LATS kinase activity with in vitro IC_50_ of 0.76 nM (LATS1) and 0.52 nM (LATS2), respectively, whereas its structurally related inactive analogue VT02484 showed no inhibition towards LATS (Fig. 5b, c). In vitro profiling against the kinome indicated that LATS1/2 were among the top hits along with the closely related NDR1/2 kinases^45^ (Supplementary Table 1).

VT02956, but not VT02484, reduced YAP/TAZ phosphorylation in both dose- and time-dependent manner with IC_50_ of 0.16 μM and 0.43 μM in HEK293A cells and 4T1 cells, respectively (Fig. 5d–f and Supplementary Fig. 7b). The apparently higher IC_50_ in cellular YAP phosphorylation assay compared to the in vitro kinase assay could be in part due to the fact that partial LATS inhibition is insufficient to reduce YAP phosphorylation in vivo^8,46^. The effect of VT02956 on YAP/TAZ activation was confirmed by qPCR analysis for YAP/TAZ target genes (Fig. 5g and Supplementary Fig. 7c, d). Importantly, we observed that VT02956 administration was accompanied with dramatic reduction of ERα and its target genes *TFF1* and *GREB1* (Fig. 5h and Supplementary Fig. 7e, f). Either *YAP/TAZ* dKO, *VGLL3* KO, or *TEAD1-4* qKO blunted the VT02956-mediated ERα downregulation (Fig. 5i and Supplementary Fig. 7g), demonstrating that the effect of VT02956 on *ESR1* is specifically mediated by LATS and YAP/TAZ.

### Pharmacological LATS inhibition suppresses ER^+^ breast cancer growth

**Fig. 6:**
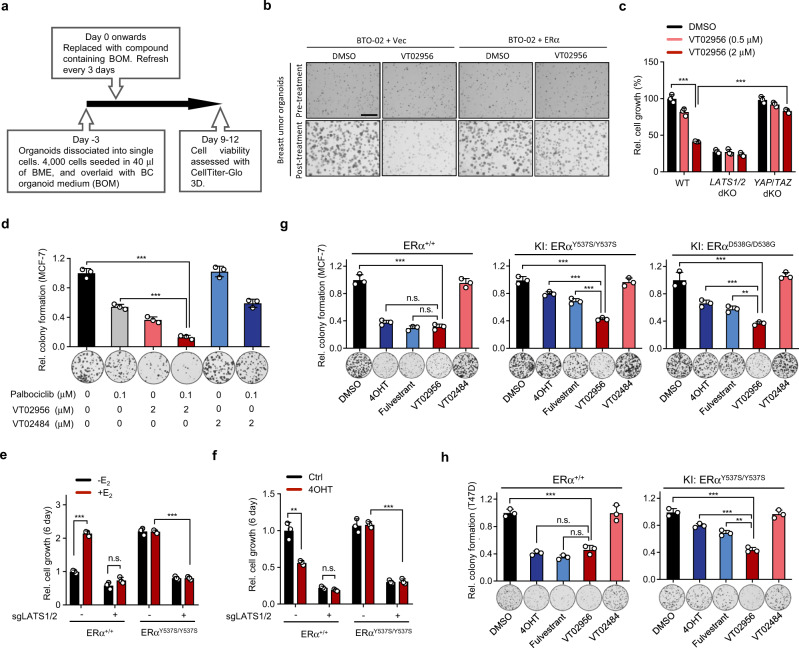
Pharmacological LATS inhibition suppresses *ER*^*+*^*breast cancer cell growth*. **a** Outline of the 3D breast tumour organoid (BTO) drug response assay. **b** Representative bright-field microscopic images of BTO-02. Tumour organoid cells were infected with lentivirus expressing ERα or control vector, and cultured in the presence of DMSO or VT02956. Pre-Treatment, at day 0 of compound treatment. Post-treatment, 12 days after compound treatment. Scale bar, 150 μm. **c** VT02956 requires LATS1/2 and YAP/TAZ to inhibit MCF-7 growth. MCF-7 cells with *LATS1/2* dKO, *YAP/TAZ* dKO, or wild-type (WT) were treatment with 0.5 μM or 2 μM VT02956 for 4 days. Cell proliferation was measured by cell counting and normalised to DMSO treated WT cells. **d** VT02956 and Palbociclib show synergistic anti-cancer effect. MCF-7 cells were treated with VT02956 (2 μM), VT02484 (2 μM) or Palbociclib (0.1 μM) alone or in combinations for 9 days. The panels show colony-formation assay stained with crystal violet from three independent experiments. **e**, **f** ERα-Y537S knock-in MCF-7 cells are resistant to 4-OHT, but still sensitive to *LATS* deletion. Fold change of cell proliferation is presented as comparison of ‒E_2_ versus +E_2_ (**e**) or DMSO versus 4-OHT (**f**). **g** VT02956 inhibits the growth of MCF-7 cells that harbour hormone therapy resistant *ESR1* mutation. MCF-7 cells with ERa Y537S or D538G knock-in were treated with 1 μM 4OHT, 0.2 μM Fulvestrant, 2 μM VT02956 and 2 μM VT02484 or DMSO for 9 days. The panel shows colony-formation assay from three independent experiments. **h** VT02956 inhibits the growth of T47D cells with hormone therapy resistant *ESR1* mutation. T47D cells with ERa Y537S knock-in were treated with 1 μM 4OHT, 0.2 μM Fulvestrant, 2 μM VT02956 and 2 μM VT02484 or DMSO for 14 days. The panel shows colony-formation assay from three independent experiments. For **c**, **d**–**h**, *n* = 3; mean ± SEM; One-way ANOVA Tukey test; n.s. not significant; **p < 0.01, ***p < 0.001; Source data are provided in the Source Data file.

We next examined whether pharmacological blockage of LATS kinase activity could inhibit the ER^+^ breast cancer cell growth. Indeed, VT02956, but not VT02484, inhibited the proliferation of MCF-7 and T47D cells, whereas VT02956 had little effect on ER^-^ cancer cells tested (Supplementary Fig. 8a–g). We posit that VT02956 inhibits the growth of ER^+^ breast cancer cells, at least in part, due to the repression of *ESR1*. Next, we performed a 3D drug killing assay using established primary ER^+^ BTOs (Fig. 6a). The growth of BTOs was sensitive to VT02956 (Fig. 6b). In addition, ectopic expression of ERα blunted the anti-growth effect of VT02956 in ER^+^ BTOs as well as in ER^+^ breast cancer cell lines (Fig. 6b and Supplementary Fig. 8h). VT02956 treatment had marginally effect on the growth of *LATS1/2* dKO nor *YAP/TAZ* dKO cells (Fig. 6c), demonstrating that VT02956 targets the LATS-YAP/TAZ-ERα axis to inhibit ER^+^ tumours cell growth.

Palbociclib, which inhibits the Cyclin-Dependent Kinase 4 and 6 (CDK4/6), is widely prescribed in combination with ERα inhibitors for treatment of ER + breast cancer^47^. We examined a combination of palbociclib and *LATS1/2* depletion, and found that such treatment dramatically reduced MCF-7 growth (Supplementary Fig. 8i). We further tested whether VT02956 would enhance the anti-tumour effect of palbociclib in ER^+^ breast cancer cells. Palbociclib or VT02956 treatment alone inhibited the colony-formation of ER^+^ breast cancer cells, whereas the combination of palbociclib and VT02956 resulted in a much more drastic reduction in colony formation (Fig. 6d and Supplementary Fig. 8j). In addition, we also tested the combinatorial effect of LATS inhibitor VT02956 and Entinostat. The data showed minor additive effect of VT02956 + Entinostat when compared to either treatment alone (Supplementary Fig. 8k), consistent with ESR1 as the common target by these two compounds.

Hormone therapy directed to reduce oestrogen levels or inhibit ERα activity is a common treatment for ER^+^ breast cancer^3^. However, patients eventually develop resistance to hormone therapy, in which more than 30% of metastatic endocrine-resistant ER^+^ breast tumours harbouring recurrent hot-spot mutations in *ESR1*^4^. Two common ERα mutation sites are Y537S/N/C and D537G, both of which are located within the ligand binding domain and confer ligand independent activity^48,49^. We asked whether targeting the LATS kinases could benefit the endocrine-resistant ER^+^ breast cancers with hot-spot mutations. We depleted *LATS1/2* in the MCF-7 cells with ERα-Y537S knock-in (KI) (Supplementary Fig. 8l). The ERα-Y537S KI cells were less sensitive to hormone depletion compared to the wild-type MCF-7 cells. In contrast, *LATS1/2* dKO decreased the cell proliferation of both ERα wild-type parental cells and ERα-Y537S KI cells (Fig. 6e). In addition, *LATS1/2* dKO inhibited the ERα-Y537S KI cells growth while 4-Hydroxytamoxifen (4-OHT), a selective oestrogen receptor modulator (SERM), was inconsequential (Fig. 6f). Next, we assessed the anti-tumour role of LATS inhibitor in ERα mutant cells and observed that VT02956 exhibited advantages over 4-OHT and Fulvestrant in inhibiting the breast cancer cells that have hormone therapy resistant hot-spot mutations, including ESR1-Y537S and ESR1-D538G (Fig. 6g, h). Thus, targeting the Hippo-ERα axis, such as using LATS inhibitors, represents a potential therapeutic approach for hormone resistant breast cancers.

## Discussion

**Fig. 7:**
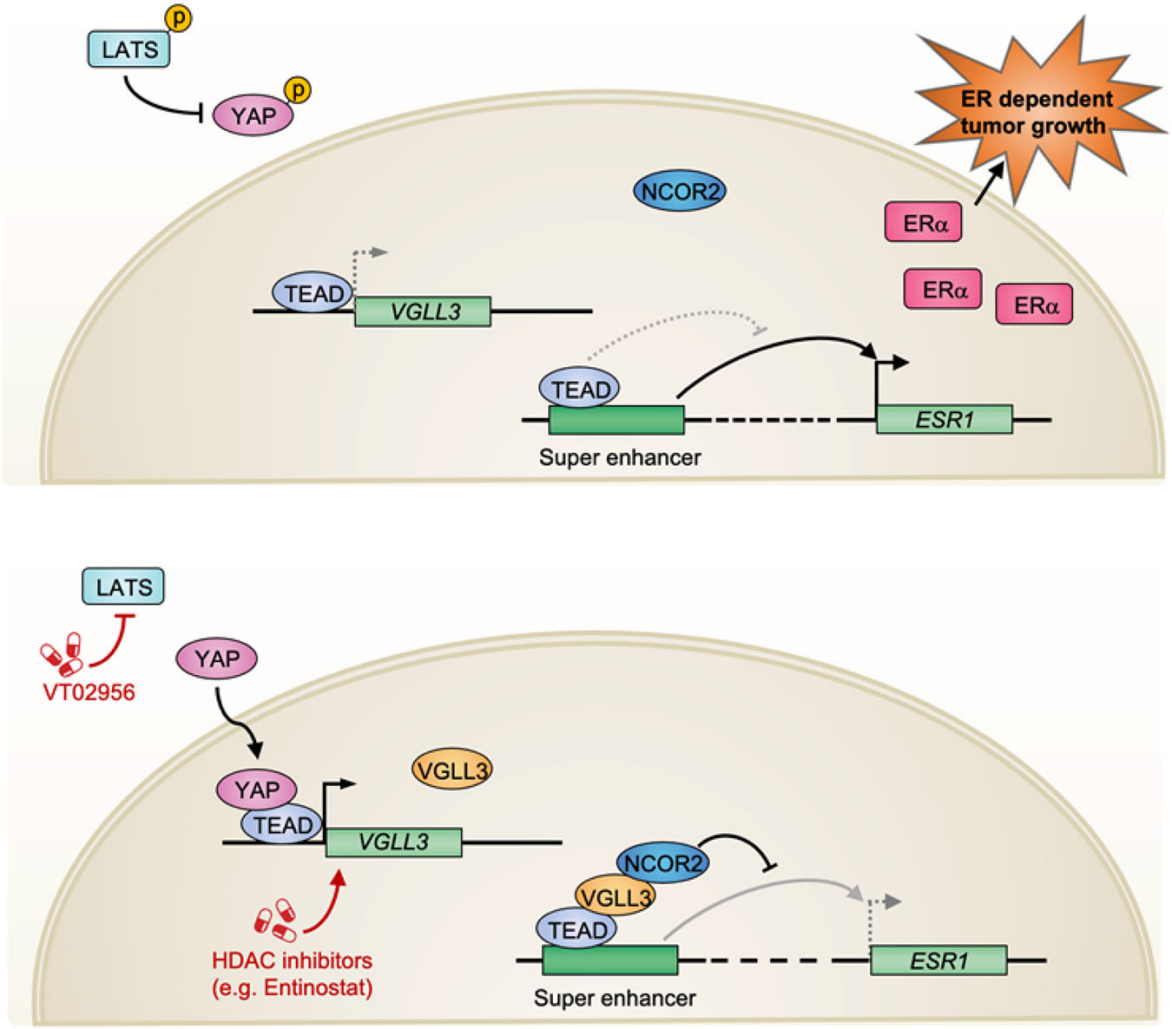
A model of the Hippo-YAP-VGLL3 axis in the regulation of *ESR1* expression and ER^+^ BRCA growth. A proposed model for ERα regulation by Hippo-YAP via VGLL3 and NCOR2. Pharmacological targeting of LATS with kinase inhibitor VT02956 or VGLL3 induction with benzamide derivative histone deacetylase (HDAC) inhibitors suppresses ERα dependent tumour growth.

In this study, we delineate a previous unrecognised signalling axis from Hippo, YAP, TEAD-VGLL3-NCOR2 to transcriptional repression of *ESR1* through epigenetic regulation of the *ESR1* super enhancer (Fig. 7), thus revealing molecular insights into the transcriptional regulation of *ESR1*. This model not only advances mechanistic understanding of *ESR1* gene repression, but also has a major implication on the Hippo pathway per se. Based on the current dogma, TEAD has low transcription activity and its activation by YAP/TAZ binding as the major functional output of Hippo signalling^8^. VGLL4 has been shown to compete with YAP/TAZ for TEAD binding^32,33^ and was proposed to inhibit YAP/TAZ by acting as a competitor, thus decreasing the transcription activity of TEAD. However, our model suggests that VGLL3 not only inhibits YAP dependent TEAD transcriptional activity, but also actively represses transcription by recruiting repressor complex to TEAD, thus revealing a feedback loop at the level of transcription. We propose that depending on the binding partners TEAD exists at least in three mutually exclusive states, active in complex with YAP/TAZ, inactive alone or in complex with VGLL1/4, and repressive in complex with VGLL2/3. Therefore, TEAD can either induce or repress target genes in a manner dependent on the associated co-factors.

We speculate that VGLL3, and possibly other VGLL family members, may play key roles in determining whether TEAD simulates or inhibits expression of downstream target genes. VGLL proteins can dislodge YAP/TAZ and recruit chromatin modifiers to genomic TEAD sites to influence transcription. Future study is needed to understand the selectivity how different VGLL members recruit different binding partners (transcription modulators) to different genomic TEAD sites, leading to specific gene regulation.

Besides NCOR2 that is required for VGLL3 to repress *ESR1*, numerous transcription regulators were identified as putative VGLL3 interacting proteins (Fig. 2a), including BCOR co-repressor, the SWI/SFN chromatin remodelling complex component ARID1A, lysine demethylase KDM1A, and histone deacetylase HDAC2. We speculate that these proteins could functionally contribute to the transcriptional regulatory activity of VGLL3, and hence TEAD. HDACs are important components of NCOR2 complex and functionally contribute to chromatin condensation and transcriptional repression^50^. Notably, we also observed that HDAC inhibitors (i.e., Entinostat) induced VGLL3 expression and repressed *ESR1* transcription (Fig. 1m, n), indicating a role of HDAC in VGLL3-induced gene regulation.

Histone deacetylase inhibitors are being actively pursued as cancer drugs^51^. Despite showing promising results in phase II clinical trials, Entinostat failed in phase III trial when in combination with exemestane, an aromatase inhibitor reducing oestrogen production, in ER positive breast cancer^36^. We show here that both Entinostat and exemestane inhibit oestrogen receptor function, thus providing a possible explanation why the two drugs do not exhibit synergy to inhibit ER^+^ breast cancer in the clinical trials. We observed that the Entinostat-mediated *ESR1* downregulation was only partially, but not completely, rescued by deletion of *VGLL3* (Fig. 1o), suggesting Entinostat has broader anti-tumour effect. HDAC inhibitors should also impede the HDAC activity within the NCOR2 repressor complex, thus potentially dampens the repression of TEAD-VGLL3-NCOR2 complex on *ESR1*. These data indicate that the VGLL3 induction might represent only one of the mechanisms by which Entinostat downregulates *ESR1*.

It was previously reported by Britschgi et al. that LATS1/2 protein induces ERα degradation independent of their kinase activity^52^. These authors further suggested that LATS functions as scaffold protein to recruit DCAF1 to induce ERα ubiquitination and degradation^52^. However, the data in this study and our recent report dispute the notion that LATS decreases ERα^24^. Britschgi et al also proposed that LATS ablation promotes luminal phenotype in normal mammary epithelial cells. However, Skibinski et al showed that TAZ overexpression or depletion in vivo results in luminal-to-basal or basal-to-luminal cell type switch, respectively^53^. The reported effects of LATS and TAZ on luminal-to-basal cell type switch by the two groups cannot be easily reconciled. Considering the multifarious and sometimes paradoxical roles of different components of the Hippo-YAP pathway in mammary epithelial cells, caution should be taken not to overinterpret the biological implications of the Hippo-YAP-VGLL3-ERa axis discovered in this study.

In the past three decades, extesive efforts have been taken to understand the tissue-specific and context-dependent expression of *ESR1*^6^. Our results support an important role of the distal super enhancer locus in the regulation of *ESR1* gene expression. Thus, it is worth to explore whether and how the *ESR1* super enhancer locus been regulated during development and tumorigenesis. In addition, what are the additional transcription factors or epigenetic regulators that control the *ESR1* super enhancer activity. Could any of them be druggable target?

The combination of LATS inhibitor VT02956 and CDK4/6 inhibitor Palbociclib suppresses MCF-7 cell growth stronger than either treatment alone, and the two inhibitors show combinatorial effect. Hormone therapy using selective oestrogen receptor modular (SERMs) or selective oestrogen receptor degraders (SERDs) is the first line treatment for ER + breast cancers, however combination with CDK4/6 inhibitor (i.e. Palbociclib) has significantly improved clinical outcome of advanced BC patients^54,55^. Our findings suggest potential therapeutic strategies of combining Entinostat with the palbociclib, particularly for endocrine-resistant BCs with ERα hot-spot mutations that are resistant to SERMs.

LATS1/2 are protein kinases and readily druggable, yet little effort has been made by the pharmaceutical industry to target LATS because they are generally considered as tumour suppressors^8^. Our data challenge this overly simplistic model, as *LATS1/2* deletion inhibits ER^+^ breast cancer cell growth due to *ESR1* transcriptional repression. In addition, we previously revealed that LATS deficiency enhances tumour cell immunogenicity^56^. Therefore, LATS may represent a therapeutic target unique for ER^+^ breast cancer and other ERα dependent cancers. In this study, we have developed a potent LATS inhibitor VT02956, which displays sub-nanomolar IC_50_ towards LATS1/2 and little cytotoxicity in cultured cells. VT02956 reduces *ESR1* expression and growth of ER^+^ breast cancer cell lines and patient-derived tumour organoids. However, the cell line models (MCF-7, T47D and ZR-75-1) and limited numbers of patient-derived ER + BC organoids (two) utilised in this study may not capture the full spectrum of the complexity and interpatient heterogeneity of the ER + breast cancers. Thus, future study with larger tumour representation is warranted to test LATS inhibitors for ERα dependent cancers, particularly those harbouring hormone therapy resistant *ESR1* mutations.

## Methods

### Animals and human tissue

All procedure of the mouse experiments performed were conducted with the approval of the Institutional Animal Care and Use Committee (IACUC) at the University of California, San Diego. *Lats1*^*fl/fl*^*Lats2*^*fl/fl*^ mice were obtained from Dr. Randy L. Johnson (MD Anderson Cancer Center, Houston, TX, USA). Mice were maintained in plastic cages with disposable bedding with no more than 5 mice per cage and under normal 12 h light/12 h dark cycle. Littermate controls or age/sex matched mice were used as indicated.

The ER positive breast tumour samples were obtained from patients undergoing surgical treatment at the Moores Cancer Center, UC San Diego Health, with the research protocol approved by Institutional Review Board. Written informed consent was obtained from all the patients. We collected five tumour samples in total. One sample failed in organoid establishment. Two organoids were ER+ at the beginning but gradually became ER negative after long term culture and passage so were not included in the study. The two successfully established ER + BC organoids and were validated by consistent ER expression and ER functionality. All the organoids related experiments were conducted before passage 12.

### Cell culture

All cells were maintained in a humidified incubator under 37 °C with 5% CO_2_. MCF-7, MDA-MB-231, B16 and HEK293A cells were cultured in DMEM (GIBCO) supplemented with 10% fetal bovine serum (FBS, GIBCO), with 1x penicillin/streptomycin (GIBCO). T47D and 4T1 cells were cultured in RPMI 1640 (GIBCO) supplemented with 10% FBS, with 1x penicillin/streptomycin. The MCF-7 cells with ESR1-Y537S or ESR1-D538G knock-in were generated using recombinant adeno-associated virus (AAV) technology. The T47D cells with ESR1-Y537S or ESR1-D538G knock-in were carried out using CRISPR-cas9 genome-editing method. The MCF-7 knock-in and T47D knock-in cells were provided by Ben Ho Park (Vanderbilt-Ingram Cancer Center) and Steffi Oesterreich (University of Pittsburgh), respectively.

### Reagents

The reagents used were as follows: Palbociclib (0.1 μM or 0.3 μM, Selleck Chemicals, Cat #S1116), 4-Hydroxytamoxifen (0.5 μM, Sigma, Cat #h7904), Trichostatin A (0.3 μM, Sigma, Cat #T8552), Entinostat (1 μM, Cayman Chemical, Cat #13284), DZNep (1 μM, Cayman Chemical, Cat #13828), 2-PCPA (200 μM, Cayman Chemical, Cat #10010494), GSK-LSD1 (1 μM, Santa Cruz, Cat #sc-490345), and 5-Aza-2′-deoxycytidine (AZA) (10 μM, Sigma, Cat #A3656). VT02956 and VT02484 were provided by Vivace Therapeutics.

### In vitro LATS1 and LATS2 kinase inhibition assays

The kinase activity of LATS1 and LATS2 were performed based on an adapted and optimised HTRF Kinase Binding assay (Cisbio). Briefly, the concentration-response curves with compound concentration range of 10,000 to 0.17 nM with 3-fold serial dilution and 1X Kinase enzymatic buffer (Cisbio), 1.2 nM LATS1 or LATS2 kinases (Signalchem), 0.5 μM biotinylation labelled YAP peptide (Vivace) were dispensed into 384-well microplates by automated digital dispenser Echo/POD with the final DMSO concentration in all wells at 0.5%. To start the enzymatic reaction, 3000 μM ATP (for LATS1) or 6000 μM ATP (for LATS2) were added using multidrop in a 10-μl volume and incubated for 90 min at 30 °C. Subsequently, 10 μl of HTRF KinEASE detection buffer (Cisbio) containing 31.25 nM acceptor conjugate (streptavidin-XL665) and 1:100 donor conjugate (TK antibody labelled with Eu^3+^-Cryptate) mixture were added and incubated at RT for 60 min, and the fluorescence emission at two different wavelengths was measured (615 nm and 665 nm) on Envision. A ratio is calculated (665/615) for each well. The inhibition was calculated by the formula, inhibition % = (HIGH − ratio) × 100/ (High − LOW). HIGH control stands for 0.5% DMSO with kinase and LOW control stands for 0.5% DMSO without kinase. The data was analysed by Xlfit software. The IC_50_ values were determined by fitting to a standard four-parameter logistic using GraphPad Prism.

### Cellular Phospho-YAP HTRF assay

Compound inhibition of in vivo YAP phosphorylation were performed based on commercial Phospho-YAP (Ser127) kit (Cisbio). In brief, HEK293A cells at 80%-100% confluence was rinsed with PBS, digested in 0.25% Trypsin-EDTA and centrifuged at 1000 × *g* for 3 min. The cell pellet was resuspended with appropriate volume of serum-free medium, counted using automated cell counter (Invitrogen), adjusted to a working density of 2.9 × 10^6^/ml and dispensed into 384-well small volume white plate (Greiner) at 12 μl/well (3.5 × 10^4^ cells) using Thermo Multidrop Combi microplate dispenser. At the time of detection, 36 nl DMSO contain 3-fold serial diluted compounds ranging from 10,000 to 4.6 nM were added into designed wells by Eco system and incubated at 37 °C for 120 min. Cell were lysed by adding 4 μl lysis buffer (4x), and incubated for 45 min at room temperature under shaking. HTRF reaction was performed by adding 4 μl of premixed phospho-YAP Cryptate antibody/Phospho-YAP d2 antibody solution prepared in the detection buffer and incubate at room temperature overnight. A ratio is calculated (665/620) for each well. The compound inhibition was calculated by the formula, inhibition % = (Ratio ZPE) × 100/ (HPE−ZPE). HPE represent 100% effect and ZPE is 0% effect. The data processing procedures were similar as in vitro LATS kinase inhibition assay.

### High-throughput compound screening

The LATS1 primary screen assay was based on HTRF kinEASE assay as mentioned above with the exception that ATP was added at the concentration of Km (31.4 μM). Out of ~17,000 compounds screened in the LATS1 HTRF kinEASE assay at single concentration of 10 μM (*n* = 1), 2422 compounds showed >50% effect and 6978 compounds showed >35% effect on TR-FRET signal. Most potent 2880 hits (ca. 47% LATS inhibition) were selected for confirmation testing (10 μM, *n* = 3). Based on re-testing results, 640 hits were selected for LATS1 IC_50_ testing (top concentration 30 μM) after excluding unacceptable structures. In all, 230 hits with LATS1 IC_50_ < 3 μM were selected for secondary screen using cell-based YAP luciferase reporter assay. Finally, 25 hits were selected for procurement (LATS1 IC_50_ < 1.5 μM; Firefly EC_50_ < 5 μM) for further validation using assays including LATS1 IP kinase assay and phosphoYAP western blot assays.

The HTS revealed the compound VT01969 has an IC_50_ = 1 μM in the LATS1 HTRF assay. Although VT01969 has not previously been reported to be a kinase inhibitor, we recognised it is an analogue of **1** and **2** (Supplimentary Fig. 7a), which have been reported to be inhibitors of atypical protein kinase C (Patent WO2014052699A9). Applying synthetic methods analogous to those reported for **1** and **2**, empirical exploration of analogues of VT01969 led to the very active compound VT02956 and the inactive compound VT02484.

### Generation and culture of mouse epithelial organoids from mammary, endometrium, fallopian tube and human breast tumour organoids

The procedure for generation and culture of mouse mammary, endometrial and fallopian tube organoids were performed as previously described^24^. The human breast tumour organoid isolation and culture were described as previously^57^ with modification. Within 2 h of surgical removal, tumour specimens were washed vigorously with Advanced DMEM/F12 containing 1x penicillin / streptomycin twice and cut into 10 mm^3^ size pieces. Two random pieces were fixed in 4% paraformaldehyde for histopathological analysis and immunohistochemistry. The remainder were finely minced with surgical scissors and digested in breast cancer (BC) organoid medium containing 2 mg/ml collagenase A (Sigma) at 37 °C for 30 min. The undigested fragments were further digested with TrypLE Express (GIBCO) at 37 °C for 10–30 min. The digested cells were washed with ice-cold phosphate-buffer saline (PBS) containing 10% fetal bovine serum (FBS) to inactivate the digestive enzymes, incubated with Red Blood Cell Lysis Buffer (Roche) to eliminate the red blood cells. The obtained breast tumour cells were embedded in Cultrex growth factor reduced BME type 2 (Trevigen) and overlaid with BC organoids medium consisting of advanced DMEM/F12, 1x GlutaMAX, 1x penicillin/streptomycin, 10 mM HEPES, 1x B-27, 5 μM Y-27632, 5 mM Nicotinamide, 1.25 mM *N*-Acetyl-l-Cysteine, 0.5 μM SB202190, 0.5 μM A83-01, 100 ng/ml Noggin, 100 ng/ml R-Spondin-1, 100 ng/ml Neuregulin-1, 5 ng/ml hEGF, 5 ng/ml FGF-7 and 20 ng/ml FGF-10.

### Immunohistochemistry

The tumour organoids were fixed with 4% paraformaldehyde in DPBS for 24 h, embedded in HistoGel before paraffin, sectioned for H&E and IHC with antibodies against ERα (1:200, CST, #8644). The signal was amplified using HRP secondary antibody, developed by ABC kit (Vectastain, PK-6100), and DAB Substrate kit (Vector Laboratories, SK-4100). The slides were counterstained with haematoxylin, dehydrated through ethanol and xylene and embedded with VectaMount medium (Vecotor Laboratories, H-5000-60). The complete slides were examined, and representative pictures was captured by Leica DMI6000 microscope and images analysed by ImageJ.

### Real-time quantitative PCR Assay

For quantitative gene expression measurement by PCR, the RNA was extracted directly from cell line samples or organoids after releasing from Matrigel by Cell Recovery Solution (Corning, #354263), followed with RNeasy Mini Kit (Qiagen, #74104). RNA was reverse transcribed into cDNA using the iScript cDNA Synthesis Kit Bundle (BioRad, #1708891). Quantitative real-time PCR (RT-qPCR) was performed by QuantStudio 3 (Applied Biosystems) with 2x KAPA SYBR FAST qPCR mix Kit (Kapa Biosystems, #KK4605). The ΔCt method was used for calculation of the relative abundance of mRNA values, which was normalised to the house-keeping transcript GAPDH as internal control. PCR primer pairs included

Human *ESR1*: left, CCCACTCAACAGCGTGTCTC; right, CGTCGATTATCTGAATTTGGCCT;

Human *TFF1*: left, CCCCGTGAAAGACAGAATTGT; right, GGTGTCGTCGAAACAGCAG;

Human *GREB1*: left, ATGGGAAATTCTTACGCTGGAC; right, CACTCGGCTACCACCTTCT;

Human *CTGF*: left, CCAATGACAACGCCTCCTG; right, TGGTGCAGCCAGAAAGCTC;

Human *CYR61*: left, AGCCTCGCATCCTATACAACC; right, TTCTTTCACAAGGCGGCACTC;

Human *ANKRD1*: left, CGTGGAGGAAACCTGGATGTT; right, GTGCTGAGCAACTTATCTCGG;

Human *VGLL1*: left, CCAAAGGCAAACAGAAGCCTA; right, CATCACACCTTCACTCTGACTC;

Human *VGLL2*: left, CCTACCACCAGAAACTAGCCT; right, GCCCTGCTGAAATGTTCATCC;

Human *VGLL3*: left, GAAGTTAGCGGTATTCAGCAAGA; right, AGCGAGAGTTAAGGTACTCCAT;

Human *VGLL4*: left, AACTGCAACCTCTCGCACTG; right, GCTCGGGCTCCTTGTAATTCT;

Human *NCOR2*: left, TGCAGATCATCTACGACGAGA; right, TCCGCATCGCCTGGTTTATTT;

Human *GAPDH*: left, GCAAATTCCATGGCACCGT; right, TCGCCCCACTTGATTTTGG;

Mouse *Esr1*: left, TGTGTCCAGCTACAAACCAATG; right, CATCATGCCCACTTCGTAACA;

Mouse *Vgll1*: left, GGTAAAGACAGAGTGGAACGC; right, GGGGCCTCTTGAGGTTACG;

Mouse *Vgll2*: left, CCACCAGAAACTAGCCTACTACT; right, ACTGAAATGTTCGTCCACCAC;

Mouse *Vgll3*: left, AGTTGTGCGGAGGTGATGTAT; right, CCGGATGATAGCAGGCTGTAG;

Mouse *Vgll4*: left, AAGATGGACCTGTTGAACTACCA; right, TTCACCTTCATAGCACAGAACG;

Mouse *Gapdh*: left, GCCTGGAGAAACCTGCCAAGTATG; right, GAGTGGGAGTTGCTGTTGAAGTCG;

### Luciferase reported assays

To assess the YAP-TEAD response of the *VGLL3* promoter locus, MCF-7 cells transfected with a pGL3-VGLL3 promoter (5.5 kb in length) firefly luciferase reporter, pCMV YAP-Flag, pRK7 TEAD4-Myc and Renilla luciferase internal control plasmid were seeded in 48-well plates and luciferase assay was performed 36 h after transfection using the Dual-Glo Luciferase Assay System (Promega, #E2920) and the firefly luciferase signal was normalised with the Renilla control.

### Generation of knock-out cells by CRISPR-cas9

Multiple gene deletion clones developed in this study were created through the CRISPR/Cas9 mediated gene editing system, and the procedure was in accordance with a previous report^24^. The targeting sequences for sgRNA used were as following: *VGLL3* sgRNA: TTAAGGTACTCCATCTCGGC; *TEAD1* sgRNA: TGGCAGTGGCCGAGACGATC; *TEAD2* sgRNA: TCTATCCACCCTGCGGCCGC; *TEAD3* sgRNA: ATGATCTTCCGCCGGCCGCA; *TEAD4* sgRNA: CTCAAGGATCTCTTCGAACG; *ESR1* sgRNA: GCCGTGTACAACTACCCCGA; *NCOR2* sgRNA: CGGTGCTTCGACTCGATGGG; *ARID1A* sgRNA: TCTCGGGGAGCTCAGCGCGT; *ZNF217* sgRNA: ACGGGCTGTCGTTCTTGGCG; *MED15* sgRNA: GGAATTGGCATGCCTCCTCG; *BCOR* sgRNA: AGTTCATCATGCCCGCGCAT; *GSE1* sgRNA: ACGTGAGCGCGAACGCGAGA; *TLE3* sgRNA: TGCCTATGGCCGATCGCCAA; *ZNF281* sgRNA: CCAGATTACCCATATTGGTA.

### Virus infection, exogenous gene expression and RNA interference

Lentivirus were generated using second-generation lentiviral system in HEK293T cells. Cell line exogenous gene expression were mediated by lentiviral infection. Briefly, HEK293T cells were co-transfected with packaging plasmids psPAX2, pMD2.g and a lentiviral transfer vector at a ratio of 3:2:5 ratio. The harvested crude virus from cultured supernatant was further purified by centrifugation followed with 0.45 μm filtration and added to the target cells with the help of 5 μg/ml polybrene. Positive selection with 1–2 μg/ml Puromycin or 200–500 μg/ml Hygromycin B in the culture medium were conducted 48 h after infection.

For lentiviral infection of breast tumour organoids cells, virus was further concentrated by centrifugation at 7,000 × *g* for 20 min at 4 °C with 8.5% polyethylene glycol 6000 and 0.3 M NaCl. Pellets were resuspended in BC organoids medium and snap frozen for later infection. The titration of lentiviral vectors was calculated through serial dilution based positive infection of HEK293A cells. Spin infection at 600 g at 32 °C for 1 h was applied for virus infection into TrypLE dissociated tumour organoids cells.

All transient transfections were performed using PolyJet DNA in vitro transfection reagent (Signagen Laboratories) in accordance with the manufacturer’s instrument. Specifically, the DNA and PolyJet reagent ratio were 1–3 μl.

For doxycycline-inducible expression assay, cell lines or tumour organoids were infected with a lentivirus encoding all-in-one constructs with improved tetracycline-controlled transactivator (TetR) and target gene expression driven by tandem Tet operators. The constructs carry puromycin resistant gene for positive selection. For the induction of target gene expression, titrated doxycycline was supplemented into the culture medium to achieve an expression of near endogenous level.

Gene silencing by RNA interference was performed with lentivirus-based shRNA. The infection process was similar as above. The shRNA constructs were generated with ligation of targeted oligonucleotide with pLKO.1 vector. An shRNA targeting LacZ (5′-AAGGCCAGACGCGAATTAT-3′), with no targeting in the human genome, was used as a control.

### Clonogenic assays and cell proliferation assays

For colony formation, cells were plated into 12-well plate with a constant density at the start date of treatment in triplicate with the indicated compounds and further cultured for 10 days (MCF-7) or 14 days (T47D). The drugs were refreshed every 2–4 days. The colonies were then fixed with 4% paraformaldehyde for 15 min and stained with 0.1% crystal violet in 20% methanol solution for quantification.

Cell proliferation rates were determined by automated cell counter. In brief, cells were plated at density of 0.1 million cells per well in six-well plate. Cell numbers were counted by the indicated time point as stated in the figure legends.

### 3D organoids drug response assays

Breast tumour organoids were dissociated into single cells using TrypLE Express and counted with automated cell counter. Subsequently, 1000 cells were mixed in 10 μl BME type 2, seeded onto 96-well plate in triplicate and overlaid with 100 μl BC organoid medium (BOM) after BME gelation. Three days after organoids sphere formation, medium was removed and replaced by 100 μl of complete medium containing VT02956 (0, 0.04, 0.2, 1, 5 μM) or vehicle (DMSO). The drug-containing medium was refreshed every 3 days for additional 3–4 times. At the end of the treatment, the effect of the drugs on the tumour organoids, in terms of the organoids cell growth, was assayed with CellTiter-Glo 3D (Promega) according to the manufacturer’s instructions and were normalised to vehicle controls (0.5% DMSO content). The IC_50_ values was calculated in GraphPad Prism 8.

### Immunoblot analysis and immunoprecipitation

Immunoblot was performed per a general western-blot protocol (Abcam). Antibodies for Flag-tag (#14793, 1:1000), HA-tag (#2367, 1:1000), Myc-tag (#2278, 1:1000), YAP (#14074, 1:1000), pYAP(S127, 1:2000) (#4911), LATS1 (#3477, 1:1000), LATS2 (#5888, 1:1000), pLATS1/2 (HM) (#8654, 1:1000), NCOR2 (#62370, 1:1000) and ERα (human) (#8644, 1:2000) were from Cell Signaling Technology. The GAPDH (#sc-25778, 1:3000), YAP/TAZ (#sc-101199, 1:2000) and ERα (mouse) (#sc-542, 1:1000) were obtained from Santa Cruz Biotechnology. The anti-Flag (HRP) (#A5892, 1:3000) was from Sigma.

For immunoprecipitations, cells were rinsed with ice-cold DPBS and then lysed in mild lysis buffer (150 mM NaCl, 50 mM Tris-Cl pH7.5, 0.5% Triton X-100) with protease inhibitors (Roche, #11873580001) and phosphatase inhibitors (Thermo Scientific, #88667) on ice for 30 min and centrifuged at 12,000 rpm for 10 min. Primary antibodies and Protein A/G magnetic beads (Pierce, #88803) were added to the supernatants and incubated with rotation for 3 h at 4 °C. Immunoprecipitants were washed four times with lysis buffer and were denatured by SDS-PAGE sample buffer and boiled for 5 min. Sample were followed by immunoblot analysis with antibodies indicated in the figures.

### TurboID affinity purification and mass spectrometry analysis

TurboID affinity purification was performed via biotin labelling and streptavidin-conjugated magnetic beads purification following the published method^30^ with modifications. Briefly, MCF-7 cells stably expressing TurboID conjugated with VGLL3, YAP or TurboID-vector were transiently labelled with 1 μM biotin for 10 min. The cells were lysed in RIPA buffer with sonication, and biotin labelled proteins were purified with streptavidin-conjugated magnetic beads (ThermoFisher) after stringent wash steps.

For mass-spectrometry analysis, the enriched proteins were digested by trypsin on-beads and proceeded by the University of California, San Diego Mass Spectrometry Core. The data analysis was carried out using the Byonic (Protein Metrics).

### RNA-seq and analysis

RNA from MCF-7 and T47D with *LATS1/2* deletion and the corresponding parental cells was prepared using the RNeasy Mini Kit as above. RNA concentration and quality was quantified by Qubit and Agilent 2100 Bioanalyzer system, respectively. Libraries were generated using NEBNext Ultra II RNA Kits (NEB) and sequenced on the NovaSeq 6000 with 100 bp paired-end reads. Following sequencing, quality control with FastQC and trimming of adaptor sequence and poly-A tails with Trim Galore, reads were mapped and assigned to the human transcripts using Salmon. Differential expression gene (DEG) analysis was performed using the DESeq2 package in R. Genes with log_2_ fold changes above 1 or below −1 and adjusted *p* value < 0.01 were considered as significantly upregulated or downregulated genes, respectively.

### Chromatin immunoprecipitation and sequencing

ChIP experiments were performed as previously described^58^. Briefly, 10–20 million cells were dual cross-linked with 1 mM disuccinimidyl glutarate (DSG, ProteoChem, #C1104-1GM) for 0.5-1 hour at room temperature and followed by 1% formaldehyde for 10 min. Cells were then incubated with 0.125 M glycine in DPBS for 5 min, collected into 1.5 ml eppendorf tube with a scraper, washed once with ice-cold DPBS, and stored in −80 °C or proceeded ahead. Cell were then lysed in 300 μl lysis buffer (5 mM Tris-HCl pH 7.8, 10 mM EDTA, 1% SDS and 1x Protease inhibitor cocktail), sonicated by Covaris water bath ultrasonicator for 2 × 30 s to achieve a final DNA fragment length between 200 and 500 bp. The supernatant was diluted 10-fold with dilution buffer (20 mM Tris-HCl pH 7.8, 2 mM EDTA, 150 mM NaCl, 1% Triton X-100 and 1x Protease inhibitor cocktail), and precleared with 10 μl Dynabeads Protein G (Invitrogen). Immunoprecipitation with anti-HA (CST, #2367, 1:200), anti-ERα (CST, #8644, 1:200), anti-Rabbit IgG (Sigma, #SAB3700889, 1:200) was performed at 4 °C overnight with constant rotation and followed with pre-cleared 25 μl protein G magnetic beads for additional 2–4 hours at 4 °C. The Immunoprecipitants were washed by once TSE1 buffer (20 mM Tris-HCl pH 7.8, 2 mM EDTA, 0.1% SDS, 1% Triton X-100), once TSE2 buffer (20 mM Tris-HCl pH 7.8, 400 mM NaCl, 2 mM EDTA, 0.1% SDS, 1% Triton X-100), twice TE buffer and resuspended in 300 μl TE/SDS buffer (1% SDS). The reverse cross-link was carried out at 65 °C for 14 h and DNA was purified with QIAquick PCR Purification Kit (Qiagen, # 28104).

The ChIP-seq DNA library construction was performed using the Rapid DNA Lib Prep Kit (ABclonal, #RK20200). Constructed libraries were further double size selected between 150-600 bp with AMPure XP (Beckman, #A63881) and sequenced on HiSeq 4000 with 75 bp single-end reads or NovaSeq 6000 with 100 bp paired-end reads at the UCSD IGM Genomics Center. The sequence tag returned by the Illumina Pipeline was adaptor trimmed by Trim Galore and aligned to the hg38 assembly by Burrows-Wheeler Aligner (BWA). The uniquely mapped reads were marked and selected by samtools view and bamtools filter, respective. The genomic binding peaks were identified using MACS2.0. The data were visualised on Easeq or IGV. Heatmap were generated by Easeq. Motif screening and analysis was performed on MEME-Suit with default setting.

### CUT&Tag-seq and analysis

Bench top CUT&Tag version 3 was performed as previously described^59^, with minor modifications. Specifically, one hundred thousand cells were harvested for each reaction with light fixation (0.1% FA for 2 min) for non-histone epitopes or without fixation for histone marks. The cells were captured by Convanavalin A-coated magnetic beads to facilitate subsequent washing steps and the reaction was carried out in 0.2 ml PCR tubes. Primary antibodies include Anti-H3K4me1 (Abcam, #ab8895, 1:200), Anti-H3K4me3 (CST, #9727, 1:200), Anti-H3K9me3 (Abcam, #ab8898, 1:200), Anti-H3K27me3 (Millipore Sigma, #07-449, 1:200), Anti-H3K27ac (CST, #8173, 1:200), Anti-H3K36me3 (CST, #4909, 1:200), Anti-H4K20me1 (Active Motif, #39727, 1:200), Anti-CTCF (CST, #3418, 1:200), Anti-HA (CST, #3724, 1:200), Normal rabbit IgG (CST, #2729, 1:400). Anti-PolII (Active Motif, #39497, 1:200), Anti-pPolII-S2 (CST, #13499, 1:200). After tagmentation, the nuclei were pelleted and de-crosslinked in lysis buffer (20 mM Tris-HCl pH 8.0, 25 mM EDTA, 0.5% SDS, 150 mM NaCl and 0.1 mg/ml Proteinase K) at 60 °C for 30 min, followed by DNA purification with DNA Clean and Concentrator-5 Kit (Zymo, #D4014). The DNA was eluted and utilised as template for library generation with PCR primers pairs similar as in the ATAC-seq, double size selected between 150 and 600 bp with AMPure XP beads, quantified by real-time qPCR and Tapestation, and sequenced on the NovaSeq 6000 with 100 bp paired-end reads at the UCSD IGM Genomics Center. The data analysis procedures were similar with in ChIP-seq above, except peak calling using SEACR^60^ optimised for the sparse nature of the CUT&Tag data.

### Assay for transposase-accessible chromatin-seq and analysis

ATAC was performed based on an optimised protocol called Omni-ATAC^61^. Briefly, 50,000 viable cells were pelleted at 4 °C and resuspended in 50 μl ice-cold ATAC-Resuspension Buffer (10 mM Tris-HCl pH 7.4, 10 mM NaCl, 3 mM MgCl_2_) supplemented with 0.01% Digitonin, 0.1% Tween-20 and 0.1% NP-40 on ice for 3 min. The lysis was then washed out once with 1 ml ice-cold ATAC-Resuspension Buffer supplemented with 0.1% Tween-20, pelleted at 500 rcf for 10 min at 4 °C, resuspended in 50 μl transposition mixture (10 mM Tris-HCl pH 7.6, 5 mM MgCl_2_, 10% Dimethyl formamide, 33% DPBS, 0.01% Digitonin, 0.1% Tween-20 and 100 nM mosaic-end incorporated Tn5 transposase), and incubated at 37 °C for 30 min with 1000 rpm vortexing. The reaction was stopped by adding DNA Binding Buffer and Tagmented DNA cleaned up with DNA Clean and Concentrator-5 Kit (Zymo, #D4014) according to the manufacturer’s protocol and eluted in 25 ul Elution Buffer (1 mM Tris-HCl pH 8.0, 0.1 mM EDTA). The ATAC library was amplified with NEBNext 2x MasterMix (non-hot start) before reaching saturation. Constructed libraries were sequenced on the NovaSeq 6000 with 100 bp paired-end reads at the UCSD IGM Genomics Center and the generated Fastq files were undergo quality control with FastQC, adaptor removal with Trim Galore, aligned into human genomes (hg38) with Bowtie2 and peak-calling by Genrich. The peak quantification and heatmap generation were performed with EaSeq.

### Multiplexing in-situ UMI-4C-seq and analysis

The 3C templates were generated by combining the protocols of 3C^40^ with in-situ Hi-C^41^. Briefly, 10 million cells were collected, and the cell pellet was resuspended in DPBS with formaldehyde added at a final concentration of 1% at room temperature for 10 min. After quenching the crosslinking reaction with 0.125 M glycine in DPBS for 5 minutes, cells were washed once by DPBS, resuspended in 300 μl in-situ lysis buffer (10 mM Tris-HCl pH 8.0, 10 mM NaCl, 0.2 5 Igepal CA-630 and 1x Protease inhibitor cocktail), and incubated on ice for 15 minutes. The nuclei were further washed once by in-situ lysis buffer and resuspended in 50 μl 0.5% SDS solution at 62 °C for exactly 10 min. The SDS was then neutralised by adding 145 μl H2O and 25 μl 10% Triton X-100 at 37 °C for 15 min. After adding 100 U DpnII (NEB, #R0543) with the presence of 1x NBEuffer 3.1, the restriction enzyme digestion was conducted in a thermomixer overnight at 37 °C. The restriction enzyme was then heat inactivated by incubating at 65 °C for 20 minutes. Then the digested chromatin was re-ligated by adding 900 μl Ligation Master Mix (1.3x NEB T4 Ligase Buffer, 1.1% X-100, 0.05% BSA and 2 U/μl T4 ligase) and incubated overnight at 16 °C with constant rotation. The nuclei were then pelleted and lysed in 300 μl De-crosslink Buffer (10 mM Tris-HCl pH 8.0, 500 mM NaCl, 1% SDS, 25 mM EDTA, 20 μg/ml proteinase K, 10 μl/ml) overnight at 65 °C. The 3C DNA was then purified with phenol-chloroform, precipitated and washed in 70% ethanol, and resuspend in Elution Buffer (1 mM Tris-HCl pH 8.0, 0.1 mM EDTA) and quantified with Nanodrop. Next, 5 μg of 3C DNA was diluted in 100 μl Elution Buffer, sonicated into an average of 300–600 bp size, followed by end-repair and A-tailing and adaptor ligation. Especially, the DNA adaptor structure was asymmetric with only one in the two annealed oligos containing PCR recognising site, which increased the specificity of 4C amplicon. The 4C libraries were generated by two nested PCR reactions with pooled US primers and pooled DS primers, followed by AmpureXP beads double-size selection. Library concentration was measured with Qubit sdDNA HS Assay Kit and further qualified with Tapestation. Pooled libraries were sequenced on the NovaSeq 6000 as mentioned above. After sequencing quality control with FastQC and trimming of adaptor sequence with Trim Galore, reads were processed in accordance with the Umi4C pipeline^40^.

### Statistics and reproducibility

Statistics was performed through Graphpad Prism v.8 and Excel. Two-tailed unpaired Student’s *t-*test was applied for statistical analyses between two groups, and one-way ANOVA for multiple comparison. Additional statistical methods are stated in the *p*-value < 0.05 were considered significant when *p < 0.05, **p < 0.01, ***p < 0.001. High-throughput sequencing assays conducted in MCF-7 cells, unless noted otherwise, were conducted once, with key experiments validated in additional ER + breast cancer cell line T47D (e.g., RNA-seq, ATAC-seq, umi-4C-seq and CUT&Tag-seq of VGLL3 for *LATS1/2* dKO or parental cells). All other experiments, including western blotting, IHC, organoids growth assay, were representative of at least three independent repeats to confirm reproducibility. Data are presented as the mean + s.d. unless otherwise noted.

### Reporting summary

Further information on research design is available in the Nature Research Reporting Summary linked to this article.

## Supplementary information

Supplementary Information

Reporting Summary

## Data Availability

The sequencing data are available at the Gene Expression Omnibus (GEO) database with accession “GSE181460”. All the other data are available within the article and its Supplementary Information. Source data are provided with this paper.

## Source data

Source Data
