# Supplementary material for: Transcriptional repression of estrogen receptor alpha by YAP reveals the Hippo pathway as therapeutic target for ER+ breast cancer
Source: Nat Commun. 2022 Feb 25;13:1061. doi: 10.1038/s41467-022-28691-0 (PMC8881512; doi:10.1038/s41467-022-28691-0)
Supplement: Supplementary file 1 — Supplementary Information [file 41467_2022_28691_MOESM1_ESM.pdf]

Supplementary information for:

Transcriptional repression of estrogen receptor alpha by YAP reveals the Hippo pathway as therapeutic target for ER$^+$ breast cancer

Shenghong Ma[1], Tracy Tang[2], Gary Probst[2], Andrei Konradi[2], Chunyu Jin[3], Fulong Li[1], J. Silvio Gutkind[1], Xiang-Dong Fu[4] and Kun-Liang Guan[1]*

[1]Department of Pharmacology and Moores Cancer Center, University of California San Diego, La Jolla, CA 92093, USA
[2]Vivace Therapeutics, San Mateo, CA 94403, USA
[3]Howard Hughes Medical Institute, Department of Medicine, University of California San Diego, La Jolla, CA 92093, USA
[4]Department of Cellular and Molecular Medicine, University of California San Diego, La Jolla, CA 92093, USA

*E-mail: kuguan@ucsd.edu

Supplementary Figure 1-8
Supplementary Table 1

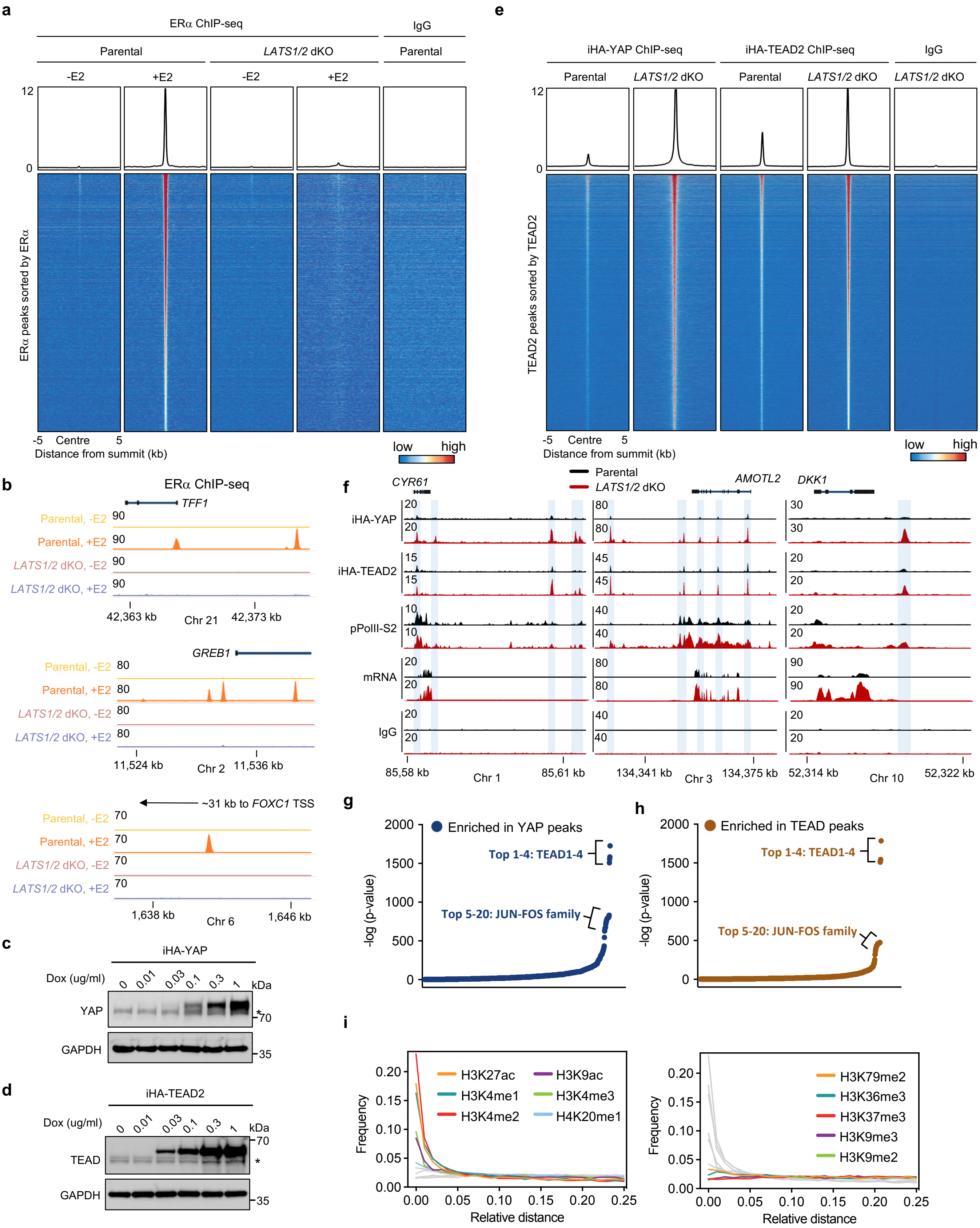
a
ERα ChIP-seq
IgG
Parental
LATS1/2 dKO
Parental
-E2
+E2
-E2
+E2
12
0
ERα peaks sorted by ERα
-5
Centre
5
Distance from summit (kb)
low
high
e
iHA-YAP ChIP-seq
iHA-TEAD2 ChIP-seq
IgG
Parental
LATS1/2 dKO
Parental
LATS1/2 dKO
LATS1/2 dKO
TEAD2 peaks sorted by TEAD2
Distance from summit (kb)
b
ERα ChIP-seq
TFF1
Parental, -E2
Parental, +E2
LATS1/2 dKO, -E2
LATS1/2 dKO, +E2
42,363 kb
Chr 21
42,373 kb
GREB1
11,524 kb
Chr 2
11,536 kb
~31 kb to FOXC1 TSS
1,638 kb
Chr 6
1,646 kb
c
iHA-YAP
Dox (ug/ml)
0
0.01
0.03
0.1
0.3
1
kDa
YAP
GAPDH
70
35
d
iHA-TEAD2
TEAD
GAPDH
f
CYR61
Parental
LATS1/2 dKO
AMOTL2
DKK1
iHA-YAP
iHA-TEAD2
pPolII-S2
mRNA
IgG
85,58 kb
Chr 1
85,61 kb
134,341 kb
Chr 3
134,375 kb
52,314 kb
Chr 10
52,322 kb
g
Enriched in YAP peaks
Top 1-4: TEAD1-4
Top 5-20: JUN-FOS family
-log (p-value)
h
Enriched in TEAD peaks
Top 1-4: TEAD1-4
Top 5-20: JUN-FOS family
-log (p-value)
i
H3K27ac
H3K9ac
H3K4me1
H3K4me3
H3K4me2
H4K20me1
Frequency
Relative distance
H3K79me2
H3K36me3
H3K37me3
H3K9me3
H3K9me2
Frequency
Relative distance

Supplementary Figure 1. Characterization of the YAP, TEAD and ERα cistromes in *LATS1/2* deficient cells.

a *LATS1/2* dKO abolishes estrogen-stimulated ERα cistrome binding. Heatmap and line graph of the ChIP-seq profiles in MCF-7 cells for ERα spanning ± 5 kb region from the summits of ERα peaks upon hormone starvation (-E_2, 3 days) and then E_2 treatment (1 hr) of parental and *LATS1/2* dKO MCF-7 cells. The ERα binding peaks between -E_2 and +E_2 treatment were 6 and 2854 for WT cells, and 123 and 195 for *LATS1/2* dKO cells, respectively. IgG denotes negative control ChIP with nonspecific IgG antibody. **b** Genome track visualization of ERα ChIP-seq between *LATS1/2* dKO and parental MCF-7 cells at the loci of ERα target genes *TFF1* (upper), *GREB1* (middle) and *FOXC1* (bottom). **c**, **d** Doxycycline dose dependently induces HA-YAP and HA-TEAD2 proteins. Immunoblot was performed with indicated antibodies. The asterisk indicates endogenous band of YAP (**c**) or TEADs (**d**). **e** Co-enrichment of the cistrome binding of YAP and TEAD proteins in *LATS1/2* dKO cells. Heatmap and line graph of ChIP-seq signal for doxycycline (dox) inducible HA-tagged YAP (iHA-YAP) and TEAD2 (iHA-TEAD2) in *LATS1/2* dKO and parental MCF-7 cells at the summits of iHA-TEAD2 peaks. **f** Genome track visualization of iHA-YAP ChIP-seq, iHA-TEAD2 ChIP-seq, phosphorylated (Serine 2) PolII (pPolII-S2) CUT&Tag-seq, and RNA-seq signal between *LATS1/2* dKO and parental MCF-7 cells at the loci of Hippo-YAP target genes *CYR61* (left), *AMOLT2* (middle) and *DKK1* (right). YAP-TEAD peaks of interest were highlighted by shaded blue. **g, h** Enrichment of TEAD motif in YAP and TEAD ChIP-seq peaks. Motif enrichment analysis for YAP (**c**) and TEAD (**d**) binding peaks against JASPAR CORE database (1404 profiles) and ordered by log(p-value). Notably, AP-1 motifs are also enriched in the YAP and TEAD peaks though less dramatic than the TEAD motif. **i** YAP-TEAD peaks are associated with active histone modifications. Accumulative plots showing the relative distances between YAP-TEAD co-enriched peaks and active histone modifications (top panel), but not enriched with repressive histone modifications (bottom panel). Source data are provided as a Source Data file.

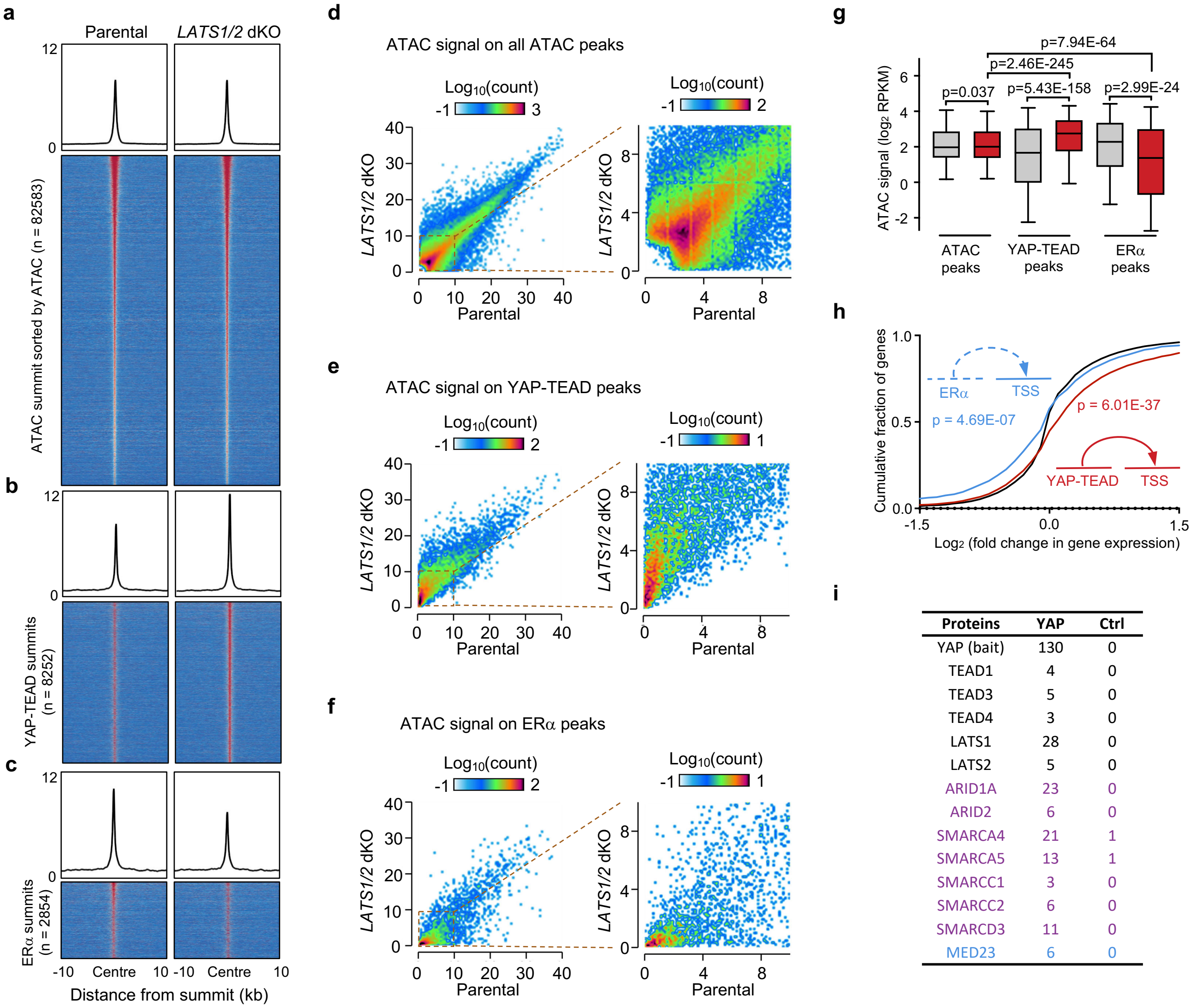

i

Proteins	YAP	Ctrl
YAP (bait)	130	0
TEAD1	4	0
TEAD3	5	0
TEAD4	3	0
LATS1	28	0
LATS2	5	0
ARID1A	23	0
ARID2	6	0
SMARCA4	21	1
SMARCA5	13	1
SMARCC1	3	0
SMARCC2	6	0
SMARCD3	11	0
MED23	6	0

Supplementary Figure 2. YAP-TEAD binding increases the chromatin accessibility and target gene expression.

a-c Heatmap and line graph comparison of ATAC-seq signals at the summits of ATAC peaks in *LATS1/2* dKO and parental MCF-7 cells (**a**), YAP/TEAD co-enriched peaks (**b**), and ERα enriched peaks (**c**). **d-f** Scatter plot showing global differences of chromatin accessibility between *LATS1/2* dKO and parental cells at the ATAC-seq peaks (**d**), YAP/TEAD co-enriched peaks (**e**), and ERα enriched peaks (**f**). **g** Increased chromatin accessibility of YAP-TEAD binding peaks in the *LATS1/2* deficient cells. Box plot for quantification of the ATAC signals between *LATS1/2* dKO and parental cells in ATAC-seq peaks, YAP/TEAD co-enriched peaks, and ERα enriched peaks. Box plot indicates median (middle line), 25-75% percentile (box), and 5-95% percentiles (whiskers). For ATAC peaks, YAP-TEAD peaks and ERα peaks, n = 82583, 8252 and 2854, respectively. Mean ± SEM; One-way ANOVA Tukey test. **** denotes $p < 0.001$. **h** YAP-TEAD binding associates with elevated gene expression. Empirical cumulative distribution function (eCDF) of the expression level changes between *LATS1/2* dKO and parental cells for YAP-TEAD connected genes (red) or ERα connected genes (blue) compared to all genes (black). Two-tailed t test. **i** YAP turboID mass spectrometry enriches TEADs, LATS, SWI/SNF complex (purple), and mediator complex (blue) components. Source data are provided as a Source Data file.

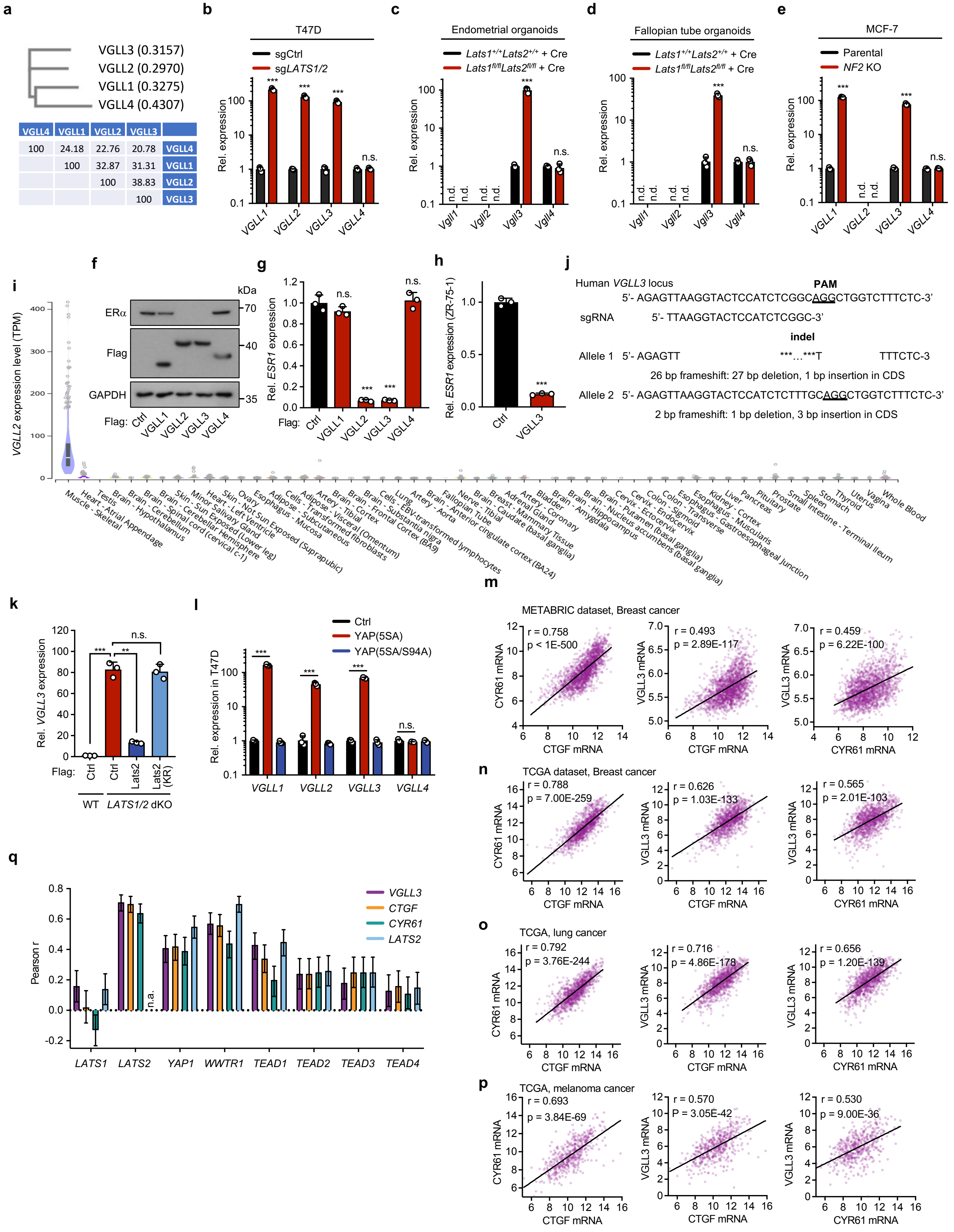
a
VGLL3 (0.3157)
VGLL2 (0.2970)
VGLL1 (0.3275)
VGLL4 (0.4307)
VGLL4 VGLL1 VGLL2 VGLL3
100 24.18 22.76 20.78 VGLL4
100 32.87 31.31 VGLL1
100 38.83 VGLL2
100 VGLL3
b
T47D
sgCtrl
sgLATS1/2
Rel. expression
VGLL1 VGLL2 VGLL3 VGLL4
n.s.
c
Endometrial organoids
Lats1+/+Lats2+/+ + Cre
Lats1fl/flLats2fl/fl + Cre
Vgll1 Vgll2 Vgll3 Vgll4
n.d.
d
Fallopian tube organoids
e
MCF-7
Parental
NF2 KO
f
ERα
Flag
GAPDH
kDa
70
40
35
Flag: Ctrl VGLL1 VGLL2 VGLL3 VGLL4
g
Rel. ESR1 expression
h
Rel. ESR1 expression (ZR-75-1)
Ctrl VGLL3
i
VGLL2 expression level (TPM)
Muscle - Skeletal
Heart - Atrial Appendage
Testis
Brain - Hypothalamus
Brain - Cerebellum
Brain - Spinal cord (cervical c-1)
Brain - Cerebellar Hemisphere
Skin - Sun Exposed (Lower leg)
Minor Salivary Gland
Heart - Left Ventricle
Skin - Not Sun Exposed (Suprapubic)
Ovary
Esophagus - Mucosa
Adipose - Subcutaneous
Cells - Transformed fibroblasts
Adipose - Visceral (Omentum)
Artery - Tibial
Brain - Cortex
Brain - Frontal Cortex (BA9)
Cells - EBV-transformed lymphocytes
Brain - Substantia nigra
Lung
Artery - Aorta
Brain - Anterior cingulate cortex (BA24)
Fallopian Tube
Nerve - Tibial
Brain - Caudate (basal ganglia)
Breast - Mammary Tissue
Adrenal Gland
Artery - Coronary
Bladder
Brain - Amygdala
Brain - Hippocampus
Brain - Nucleus accumbens (basal ganglia)
Brain - Putamen (basal ganglia)
Cervix - Ectocervix
Cervix - Endocervix
Colon - Sigmoid
Colon - Transverse
Esophagus - Gastroesophageal Junction
Esophagus - Muscularis
Kidney - Cortex
Liver
Pancreas
Pituitary
Prostate
Small Intestine - Terminal Ileum
Spleen
Stomach
Thyroid
Uterus
Vagina
Whole Blood
j
Human VGLL3 locus
PAM
5'- AGAGTTAAGGTACTCCATCTCGGCAGGCTGGTCTTTCTC-3'
sgRNA 5'- TTAAGGTACTCCATCTCGGC-3'
indel
Allele 1 5'- AGAGTT ***...***T TTTCTC-3
26 bp frameshift: 27 bp deletion, 1 bp insertion in CDS
Allele 2 5'- AGAGTTAAGGTACTCCATCTCTTTGCAGGCTGGTCTTTCTC-3'
2 bp frameshift: 1 bp deletion, 3 bp insertion in CDS
k
Rel. VGLL3 expression
Flag: Ctrl Ctrl Lats2 Lats2 (KR)
WT LATS1/2 dKO
l
Ctrl
YAP(5SA)
YAP(5SA/S94A)
Rel. expression in T47D
m
METABRIC dataset, Breast cancer
r = 0.758
p < 1E-500
r = 0.493
p = 2.89E-117
r = 0.459
p = 6.22E-100
CYR61 mRNA
VGLL3 mRNA
CTGF mRNA
n
TCGA dataset, Breast cancer
r = 0.788
p = 7.00E-259
r = 0.626
p = 1.03E-133
r = 0.565
p = 2.01E-103
o
TCGA, lung cancer
r = 0.792
p = 3.76E-244
r = 0.716
p = 4.86E-178
r = 0.656
p = 1.20E-139
p
TCGA, melanoma cancer
r = 0.693
p = 3.84E-69
r = 0.570
P = 3.05E-42
r = 0.530
p = 9.00E-36
q
Pearson r
VGLL3
CTGF
CYR61
LATS2
n.a.
LATS1 LATS2 YAP1 WWTR1 TEAD1 TEAD2 TEAD3 TEAD4

Supplementary Figure 3. VGLL3 in the Hippo-ER axis.

a Phylogenetic tree analysis showing protein sequence similarity among VGLL1-4 using Clustal Omega algorithm. Lower panel, pairwise percent identity matrix among VGLL1-4 proteins. **b** *LATS1/2* were deleted by lentivirus mediated CRISPR deletion (transient deletion pool) in T47D cells. *VGLLs* gene expression was determined by qPCR analysis. n.s., not significant. **c, d** *LATS1/2* deletion increases *VGLL3* expression in mouse endometrial organoids (**c**) and fallopian tube organoids (**d**). Organoids derived from the mouse endometrial and fallopian tube tissues of *Lats1*$^{+/+}$*Lats2*$^{+/+}$ and *Lats1*$^{fl/fl}$*Lats2*$^{fl/fl}$ mice were infected with Cre-encoding adenovirus to delete *LATS1/2*. *VGLL1-4* expression was determined by qPCR analysis. n.d., not detectable. **e** *NF2* deletion upregulates *VGLL1* and *VGLL3* expression. **f**, **g** T47D cells expressing vector control, Flag-VGLL1, VGLL2, VGLL3, or VGLL4 were subjected to immunoblot with indicated antibodies (**f**) or qPCR analysis for *ESR1* (**g**). **h** ZR-75-1 cells expressing vector control, or VGLL3 were subjected to qPCR analysis for *ESR1* mRNA. **i** *VGLL2* expression level among different tissues/organs. Data were extracted from GTEx dataset. TPM, Transcripts Per Million. Box plot indicates median (middle line), 25-75% percentile (box), data above or below 1.5 times the interquartile range are displayed as outliers. **j** Sanger DNA sequencing confirmed the *VGLL3* frameshift mutation in *VGLL3* KO MCF-7 cells generated by CRISPR/Cas9. **k** MCF-7 cells with *LATS1/2* deficiency were infected with lentiviruses encoding murine wild-type Lats2, kinase inactive mutant Lats2 (KR) or vector control. *VGLL3* expression levels were measured by qPCR. **l** T47D cells stably expressing a control vector, YAP(5SA), or YAP(5SA/S94A) were subjected to qPCR analysis for VGLL family genes. **m**-**p** Dot plots showing the positive correlation of mRNA expression levels of *VGLL3* and the known YAP target gene *CTGF* or *CYR61* in METABRIC breast cancers dataset (n = 1904) (**m**), TCGA breast cancers (n = 1218) (**n**), TCGA lung cancers (n = 1129) (**o**), and TCGA melanoma cancers (n = 477) (**p**). Pearson's correlation coefficient (r) with p value (two-tailed). **q** Pearson correlation between LATS/YAP/TEADs and VGLL3/CTGF / CYR61/LATS2 in Luminal A/B subtype breast cancers. The expression data were extracted from TCGA dataset (n = 346). The bar plot represents Pearson's correlation coefficient (r) with error bar representing 95% confidence. For **b-e**, **g-h** and **k-l**, n = 3 with mean ± SEM. Two-sided, unpaired t-test for **b-e**, **h**; one-way ANOVA Tukey test for **g, k-l**. n.d., not detectable; n.s., not significant; **p<0.01, ***p<0.001; Source data are provided as a Source Data file.

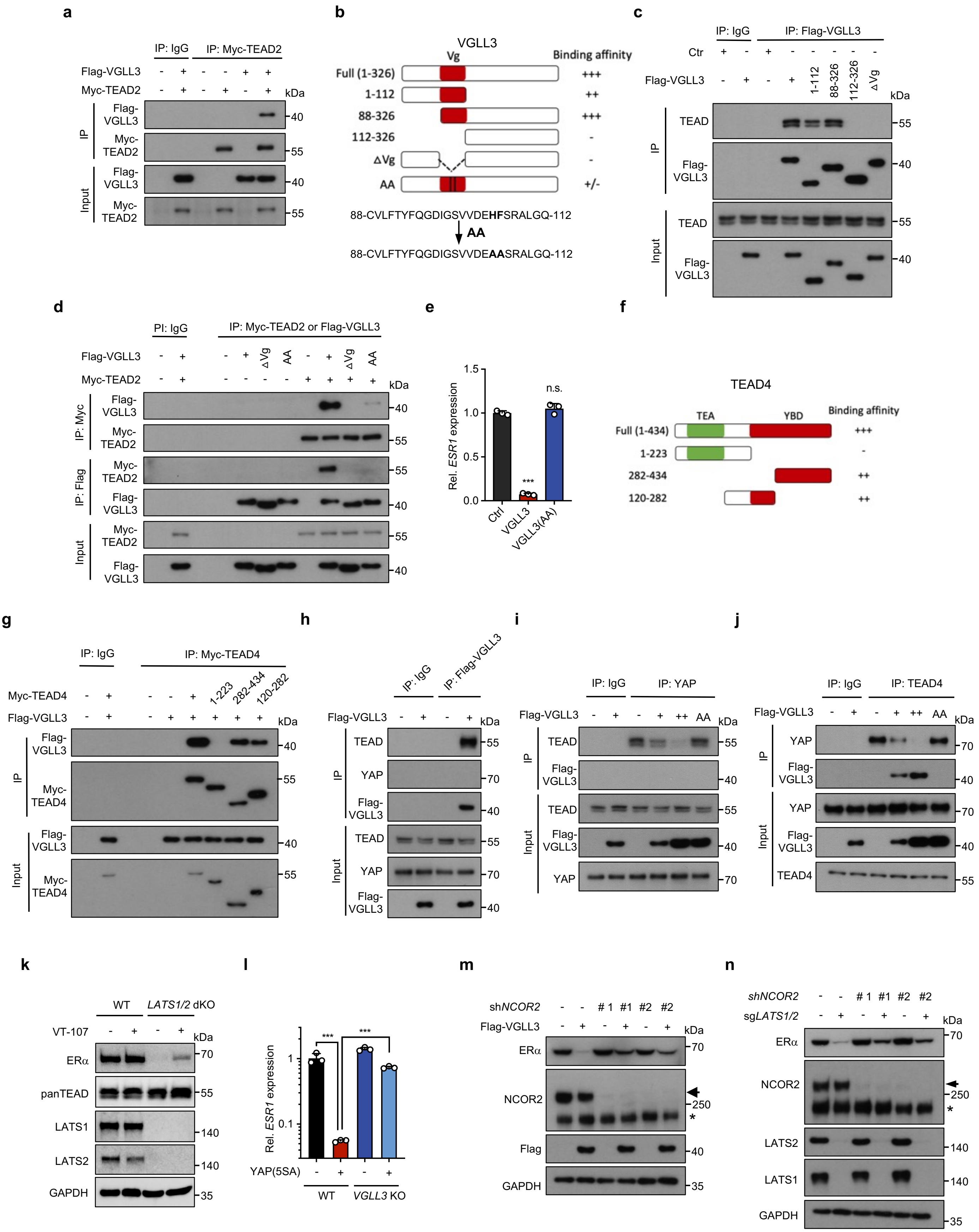
a
IP: IgG
IP: Myc-TEAD2
Flag-VGLL3
Myc-TEAD2
kDa
IP
Input
Flag-VGLL3
Myc-TEAD2
40
55
b
VGLL3
Vg
Binding affinity
Full (1-326)
1-112
88-326
112-326
ΔVg
AA
+++
++
+++
-
-
+/-
88-CVLFTYFQGDIGSVVDEHFSRALGQ-112
AA
88-CVLFTYFQGDIGSVVDEAASRALGQ-112
c
IP: IgG
IP: Flag-VGLL3
Ctr
Flag-VGLL3
1-112
88-326
112-326
ΔVg
kDa
IP
Input
TEAD
Flag-VGLL3
55
40
d
PI: IgG
IP: Myc-TEAD2 or Flag-VGLL3
Flag-VGLL3
Myc-TEAD2
ΔVg
AA
kDa
IP: Myc
IP: Flag
Input
Flag-VGLL3
Myc-TEAD2
40
55
e
Rel. ESR1 expression
1.5
1.0
0.5
0.0
n.s.
***
Ctrl
VGLL3
VGLL3(AA)
f
TEAD4
TEA
YBD
Binding affinity
Full (1-434)
1-223
282-434
120-282
+++
-
++
++
g
IP: IgG
IP: Myc-TEAD4
Myc-TEAD4
Flag-VGLL3
1-223
282-434
120-282
kDa
IP
Input
Flag-VGLL3
Myc-TEAD4
40
55
h
IP: IgG
IP: Flag-VGLL3
Flag-VGLL3
kDa
IP
Input
TEAD
YAP
Flag-VGLL3
55
70
40
i
IP: IgG
IP: YAP
Flag-VGLL3
++
AA
kDa
IP
Input
TEAD
Flag-VGLL3
YAP
55
40
70
j
IP: IgG
IP: TEAD4
Flag-VGLL3
++
AA
kDa
IP
Input
YAP
Flag-VGLL3
TEAD4
70
40
55
k
WT
LATS1/2 dKO
VT-107
kDa
ERα
panTEAD
LATS1
LATS2
GAPDH
70
55
140
140
35
l
Rel. ESR1 expression
1
0.1
***
***
YAP(5SA)
WT
VGLL3 KO
m
shNCOR2
1
#1
#2
#2
Flag-VGLL3
kDa
ERα
NCOR2
Flag
GAPDH
70
250
*
40
35
n
shNCOR2
1
#1
#2
#2
sgLATS1/2
kDa
ERα
NCOR2
LATS2
LATS1
GAPDH
70
250
*
140
140
35

Supplementary Figure 4. VGLL3 in the Hippo-ER axis.

a TEAD interacts with VGLL3. MCF-7 cells were transiently transfected with plasmids expressing the indicated proteins. Protein-protein interaction was examined by IP-western blot. **b** Mapping the TEAD binding domain in VGLL3. This is a schematic summary of truncation/mutation and co-immunoprecipitation data. **c** The Vg domain in VGLL3 is responsible for TEAD binding. Wild-type Flag-VGLL3 and its truncation mutants were transiently expressed in MCF-7 cells, immunoprecipitated with Flag antibody, and followed by western blot to detect interaction with endogenous TEADs. **d** VGLL3(AA) mutant diminishes the interaction with TEAD2. Wild-type or mutant Flag-VGLL3 were co-expressed with Myc-TEAD2 in MCF-7 cells. Protein-protein interaction was examined by IP-western. AA mutant represents H105A/F106A mutations within the Vg domain (see panel **b**). **e** VGLL3(AA) mutant is inactive to repress *ESR1*. MCF-7 cells expressing a control vector (Ctrl), VGLL3 cDNA, or VGLL3(AA) cDNA were subjected to qPCR analysis for *ESR1*. **f** Mapping the VGLL3 binding domain in TEAD4. This is a schematic summary of truncation co-immunoprecipitation data. **g** The YAP binding domain (YBD) in TEAD4 is responsible for VGLL3 binding. Wild-type Myc-TEAD4 or its deletion mutants were co-expressed with Flag-VGLL3 in MCF-7 cells. Protein-protein interaction was examined by IP-western. **h** VGLL3 does not bind YAP. **i**, **j** VGLL3 competes with YAP for TEAD binding. MCF-7 transfected with different doses of VGLL3 proteins were subjected to YAP (**i**) or TEAD (**j**) co-immunoprecipitation. Immunoblot analysis was performed with the indicated antibodies. **k** TEAD inhibitor VT-107 ameliorates the ERα reduction caused by *LATS1/2* dKO. WT or *LATS1/2* dKO MCF-7 cells treated with 1 μM of VT-107 or DMSO for 2 days and subjected for immunoblot with indicated antibodies. **l** *VGLL3* KO block YAP(5SA) mediated *ESR1* downregulation. *VGLL3* KO and parental WT MCF-7 cells transduced with YAP(5SA), or control vector were subjected to qPCR for *ESR1* expression. **m**, **n** NCOR2 knock-down blocks ERα repression by the Hippo-VGLL3 axis. MCF-7 cells transfected with different *NCOR2* shRNAs and Flag-VGLL3 cDNA (**m**) or CRISPR sgRNA targeting *LATS1/2* (**n**) were lysed for immunoblot with indicated antibodies. The asterisk indicates the nonspecific band detected by the NCOR2 antibody. For **e** and **l**, n = 3 with mean ± SEM, one-way ANOVA Tukey test; n.s., not significant; ***p<0.001; Source data are provided as a Source Data file.

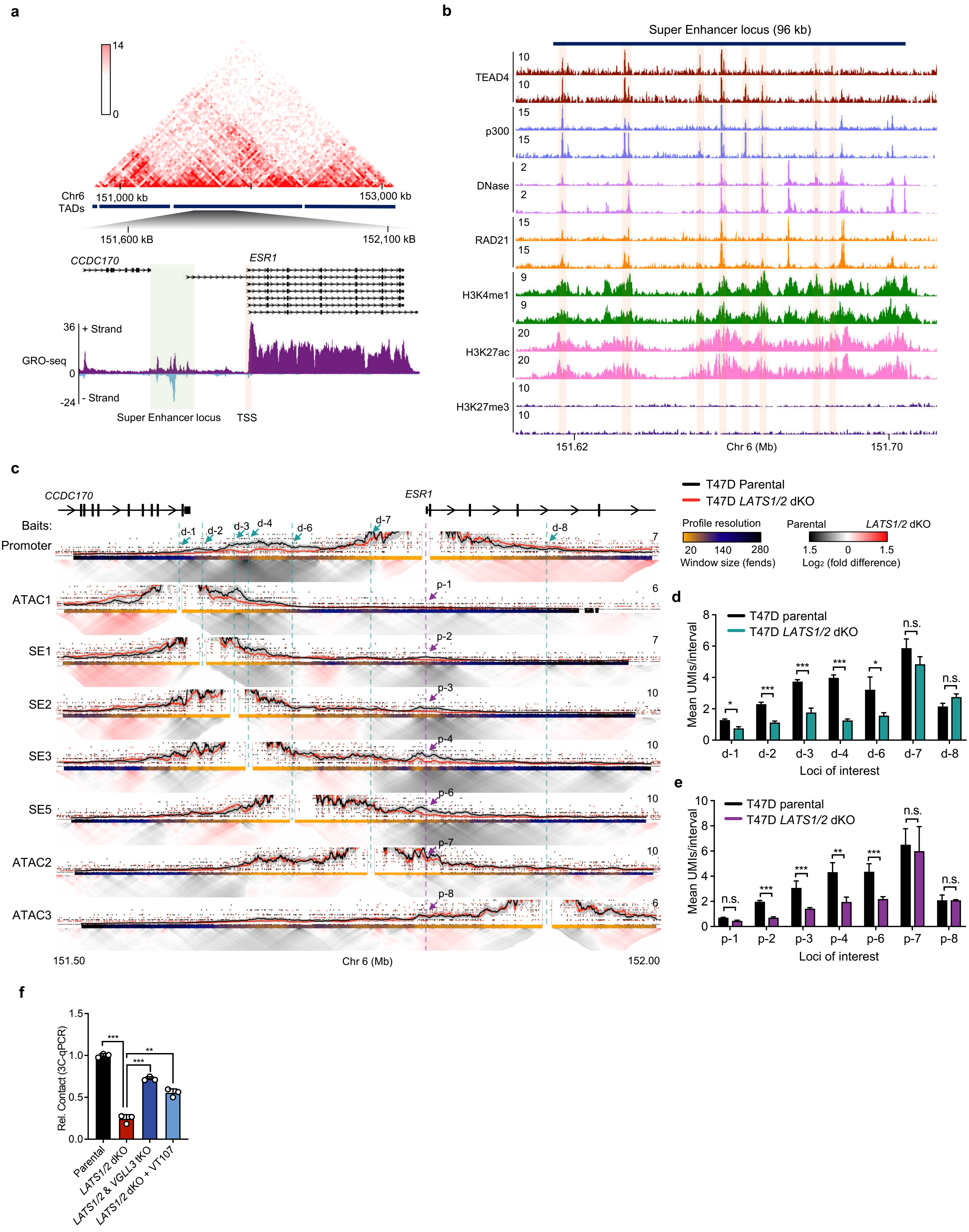

a
b
Super Enhancer locus (96 kb)
TEAD4
p300
DNase
RAD21
H3K4me1
H3K27ac
H3K27me3
151.62
Chr 6 (Mb)
151.70
Chr6
TADs
151,000 kb
153,000 kb
151,600 kB
152,100 kB
CCDC170
ESR1
+ Strand
GRO-seq
- Strand
Super Enhancer locus
TSS
c
T47D Parental
T47D LATS1/2 dKO
Profile resolution
Window size (fends)
Parental
LATS1/2 dKO
Log2 (fold difference)
Baits:
Promoter
ATAC1
SE1
SE2
SE3
SE5
ATAC2
ATAC3
151.50
Chr 6 (Mb)
152.00
d
T47D parental
T47D LATS1/2 dKO
Mean UMIs/interval
Loci of interest
e
T47D parental
T47D LATS1/2 dKO
Mean UMIs/interval
Loci of interest
f
Rel. Contact (3C-qPCR)
Parental
LATS1/2 dKO
LATS1/2 & VGLL3 tKO
LATS1/2 dKO + VT107

Supplementary Figure 5. Regulation of *ESR1* super enhancer by Hippo-ER axis.
a The *ESR1* coding region and the distal super enhancer are localized in the same topologically associated domain (TAD). Hi-C contacts maps (upper) shown the same TAD localization of the putative super enhancer locus and *ESR1* gene promoter, as defined by GRO-seq (bottom). Hi-C contact maps for human MCF-7 cells were derived from ENCODE database with 25 kb resolution. Green shaded area marks the super enhancer locus whereas yellow shaded area indicates the transcriptional start site (TSS) of the *ESR1* gene. **b** TEAD binds to the *ESR1* distal super enhancer. ChIP-seq tracks for TEAD4, P300, RAD21, H3K4me1, H3K27ac and H3K27me3, as well as DNase-seq in the *ESR1* super-enhancer locus in MCF-7 cells. TEAD4 peaks of interest were highlighted with shaded orange. Datasets were extracted from ENCODE project, each with two replications. **c** Multiplexing in-situ UMI-4C profile analysis of the *ESR1* locus between *LATS1/2* deficient (red) and parental (black) T47D cells. Domainogram colour ($\log_2$ fold difference) is relative to the maximum profile to the presented genomic window. SE1-5 represent different TEAD-VGLL3 positive peaks within the *ESR1* super enhancer locus whereas ATAC1-3 are putative distal regulatory elements outside the super enhancer. **d, e** Deletion of *LATS1/2* dampens the interaction between *ESR1* promoter and the super enhancer locus in T47D cells. Fold change of the contact intensities between *ESR1* promoter locus with each of the seven genomic intervals (denoted as d-1 to d-8, except d-5) using *ESR1* promoter locus (**d**) or seven genomic intervals as 4C baits (**e**). Error bars estimated binomial s.d.; n.s., not significant. **f** *VGLL3* KO or TEAD inhibitor ameliorates the reduced contact between *ESR1* promoter and super enhancer locus in *LATS1/2* dKO cells. MCF-7 cell with *LATS1/2* dKO alone, or in combination with *VGLL3* KO or 1 μM VT107 treatment for 2 days were harvested for 3C library construction and followed by targeted bait qPCR between *ESR1* promoter and super enhancer. For **f**, n = 3 with mean ± SEM; one-way ANOVA Tukey test; **p<0.01, ***p<0.001; Source data are provided as a Source Data file.

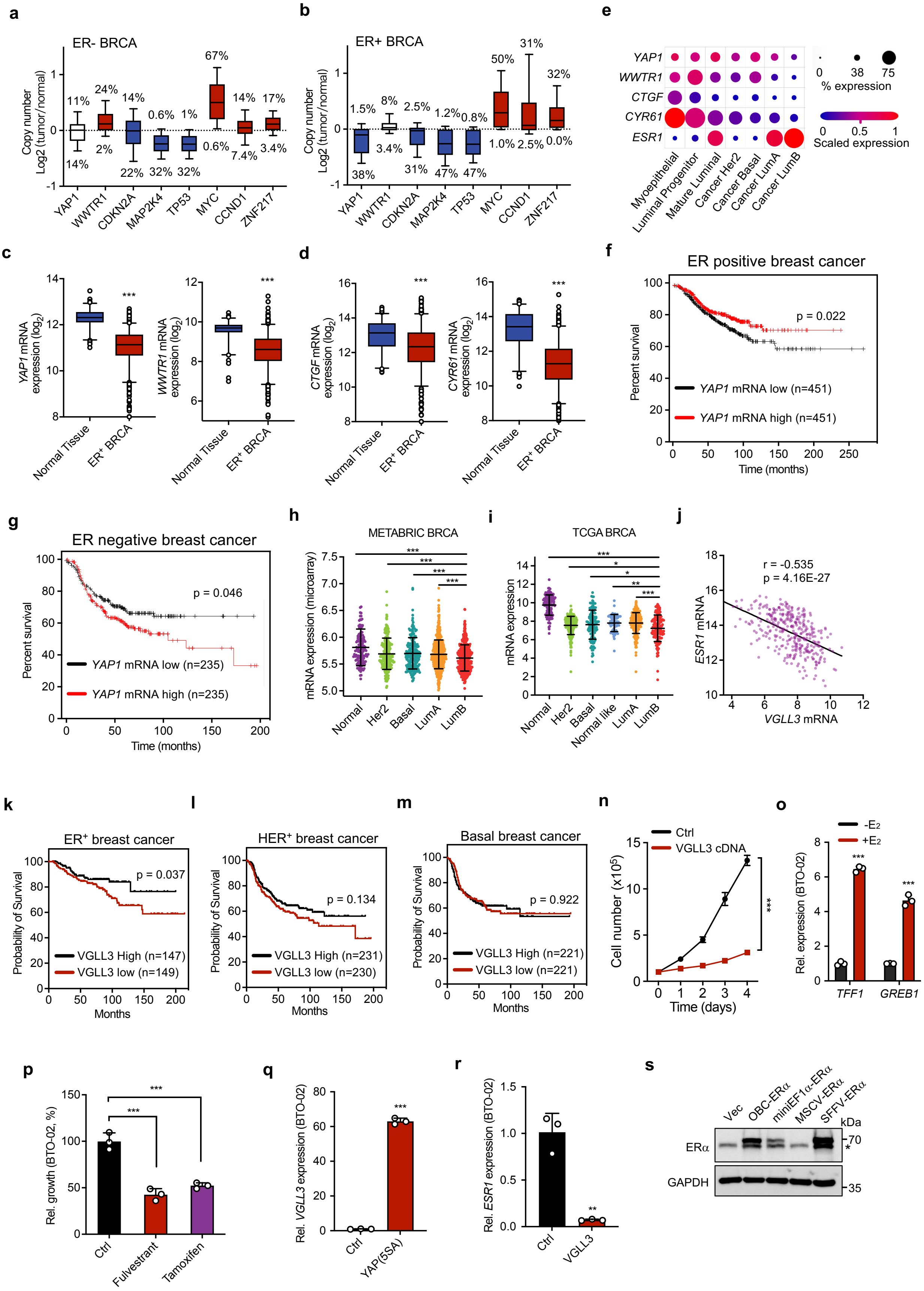

a
ER- BRCA
Copy number Log2 (tumor/normal)
YAP1
WWTR1
CDKN2A
MAP2K4
TP53
MYC
CCND1
ZNF217
b
ER+ BRCA
c
Normal Tissue
ER+ BRCA
d
e
YAP1
WWTR1
CTGF
CYR61
ESR1
Myoepithelial
Luminal Progenitor
Mature Luminal
Cancer Her2
Cancer Basal
Cancer LumA
Cancer LumB
% expression
Scaled expression
f
ER positive breast cancer
p = 0.022
YAP1 mRNA low (n=451)
YAP1 mRNA high (n=451)
Percent survival
Time (months)
g
ER negative breast cancer
p = 0.046
YAP1 mRNA low (n=235)
YAP1 mRNA high (n=235)
h
METABRIC BRCA
mRNA expression (microarray)
Normal
Her2
Basal
LumA
LumB
i
TCGA BRCA
mRNA expression
Normal like
j
r = -0.535
p = 4.16E-27
ESR1 mRNA
VGLL3 mRNA
k
ER+ breast cancer
p = 0.037
VGLL3 High (n=147)
VGLL3 low (n=149)
Probability of Survival
Months
l
HER+ breast cancer
p = 0.134
VGLL3 High (n=231)
VGLL3 low (n=230)
m
Basal breast cancer
p = 0.922
VGLL3 High (n=221)
VGLL3 low (n=221)
n
Ctrl
VGLL3 cDNA
Cell number (x10^5)
Time (days)
o
-E2
+E2
Rel. expression (BTO-02)
TFF1
GREB1
p
Rel. growth (BTO-02, %)
Ctrl
Fulvestrant
Tamoxifen
q
Rel. VGLL3 expression (BTO-02)
YAP(5SA)
r
Rel. ESR1 expression (BTO-02)
VGLL3
s
Vec
OBC-ERα
miniEF1α-ERα
MSCV-ERα
SFFV-ERα
kDa
ERα
GAPDH
70
35

Supplementary Figure 6. Hippo-ER axis in ER$^+$ patients.

a, **b** *YAP1* copy number was frequently decreased in ER$^+$ (**b**) but not ER$^-$ (**a**) invasive breast carcinoma (BRCA). Plot of chromosomal gain and loss for the selected loci, data from TCGA-BRCA, n = 176 (**a**) and 596 (**b**). The number above or below box plot represents the percentage of patients with gain-of-copy or loss-of-copy in tumours, respectively. **c, d** Box plot for the expression of *YAP1* and *WWTR1* (**c**), *CTGF* and *CYR61* (**d**) mRNA in normal tissues and ER$^+$ tumours, data from TCGA-BRCA [normal (n = 114) versus tumours (n = 596)]. The middle line represents the median gene expression levels. **e** The expression level of *YAP, WWTR1, CTGF, CYR61 and ESR1* were compared among different subtypes of cells using a published scRNA breast cancer dataset. **f**, **g** Low YAP expression was associated with poor survival in ER$^+$, but not in ER$^-$ BRCA. Kaplan-Meier curve of patient survival analysis. ER$^+$ breast cancer patients (**f**) or ER$^-$ breast cancer patients (**g**) are stratified by *VGLL3* expression levels, with the *VGLL3* high group defined as patients with more than the median *VGLL3* expression. Significance was determined by log rank analysis. **h**, **i** Expression analysis of *VGLL3* among different subtypes of breast cancers. *VGLL3* expression level were extracted from METABRIC breast cancer dataset (**h**) and TCGA breast cancer dataset (**i**), error bar SEM. **j** Dot plots showing the negative correlation of mRNA expression levels of *VGLL3* and *ESR1* in Luminal type A/B breast cancers (n = 347). Data were extracted from The Cancer Genome Atlas dataset. Pearson's correlation coefficient (r) with p value (two-tailed). **k-m** Kaplan-Meier curve of patient survival analysis. ER$^+$ (**k**), HER$^+$ (**l**), or Basal type (**m**) breast cancer patients are stratified by *VGLL3* expression levels, with the *VGLL3* high group defined as patients with more than the median *VGLL3* expression. Significance was determined by log rank analysis. **n** MCF-7 expressing a control vector (Ctrl) or *VGLL3* cDNA cells were seeded at 0.1 million cells per 6-well, cell number were counted at the indicated time points. **o** qPCR analysis of indicated genes for BTO-02 organoids starved for E_2 for 3 days and then treated with or with 100 nM E2 (+E_2 vs -E_2) for 2 hours. **p** 3D-cell growth was measured for organoids BTO-02 after 12 days treatment with 0.2 μM Fulvestrant or 1 μM Tamoxifen. **q** qPCR analysis of *VGLL3* in BTO-02 transduced with control vector or constitutively active YAP(5SA). **r** VGLL3 suppresses *ESR1* expression in breast tumour organoids. qPCR analysis of *ESR1* in BTO-02 transduced with control vector or VGLL3. **s** BTO-02 infected with lentivirus encoding different promoter-ERα-Flag constructs were lysed and subjected to immunoblot with indicated antibodies. The miniEF1-ERα produced a near endogenous ERα expression. The asterisk indicates endogenous band of ERα. Box plot in **a-d** indicates median (middle line), 25-75% percentile (box), and 10-90% percentiles (whiskers). For **o-r**, n = 3 with mean ± SEM. Two-sided, unpaired t-test for **c-d**, **o**, **q-r**; Two-way (**n**) or one-way (**p**) ANOVA Tukey test; log-rank (Mantel-Cox) test for **f-g**, **k-m**; *p<0.05, **p<0.01, ***p<0.001; Source data are provided as a Source Data file.

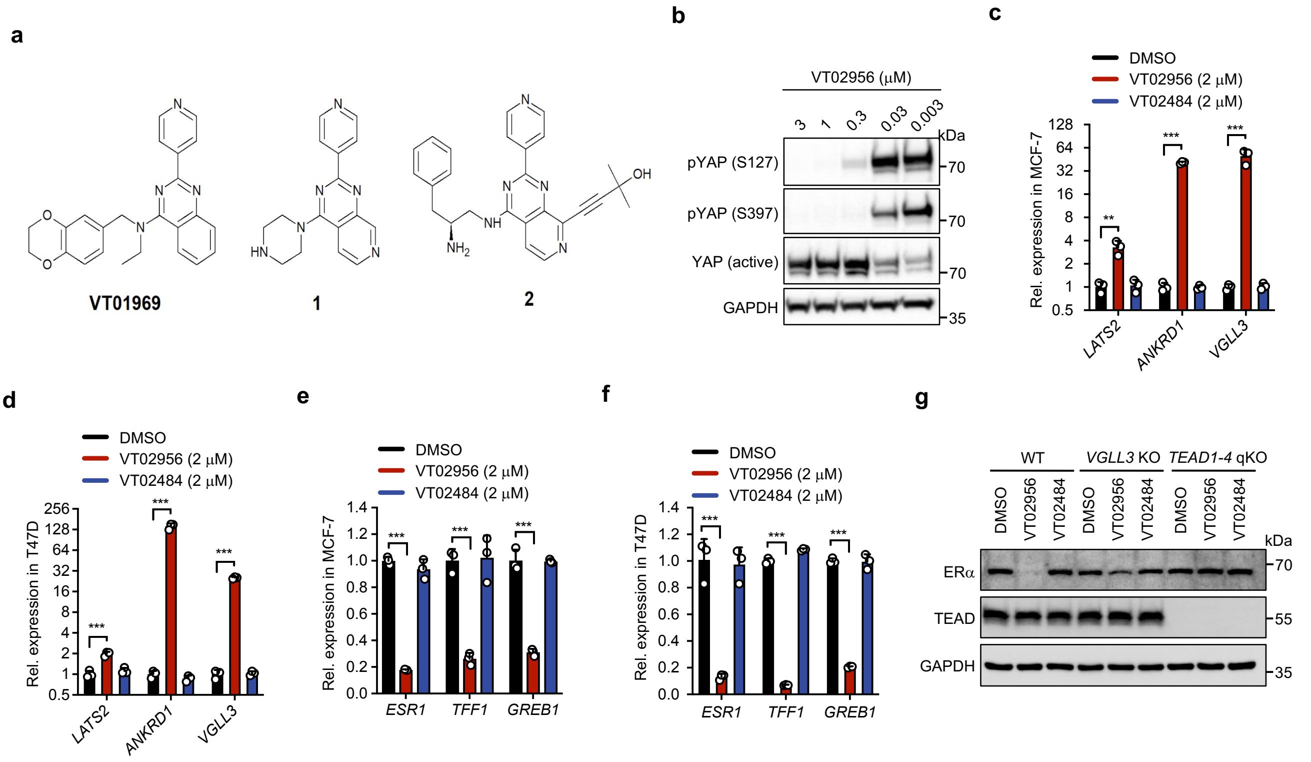
a
VT01969
1
2
b
VT02956 (μM)
3
1
0.3
0.03
0.003
kDa
pYAP (S127)
pYAP (S397)
YAP (active)
GAPDH
70
70
70
35
c
DMSO
VT02956 (2 μM)
VT02484 (2 μM)
Rel. expression in MCF-7
LATS2
ANKRD1
VGLL3
d
DMSO
VT02956 (2 μM)
VT02484 (2 μM)
Rel. expression in T47D
LATS2
ANKRD1
VGLL3
e
DMSO
VT02956 (2 μM)
VT02484 (2 μM)
Rel. expression in MCF-7
ESR1
TFF1
GREB1
f
DMSO
VT02956 (2 μM)
VT02484 (2 μM)
Rel. expression in T47D
ESR1
TFF1
GREB1
g
WT
VGLL3 KO
TEAD1-4 qKO
DMSO
VT02956
VT02484
kDa
ERα
TEAD
GAPDH
70
55
35

Supplementary Figure 7. VT02956 efficiently inhibits LATS activity and reduces *ESR1* expression.

a The structure of the initial hit VT01969 from the LATS kinase HTS screening and the analogous of 1 and 2. Details see Methods. **b** VT02956 inhibits the LATS phosphorylation in YAP. HEK293A cell treated with increased concentration of VT02956 were subjected to immunoblot with indicated antibodies recognizing YAP serine 127 phosphorylation (S127), serine 397 phosphorylation (S397), or the unphosphorylated serine 127 YAP (active). **c**, **d** VT02956 induces the expression of YAP target genes in breast cancer cell lines. qPCR analysis of selected genes in MCF-7 (**c**) or T47D cells (**d**) treated with 2 μM VT02956, 2 μM VT02484 or DMSO control for 2 days. **e**, **f** VT02956 suppresses the expression of *ESR1* and ERα target genes in MCF-7 cells (**e**) and T47D cells (**f**). Experiments were conducted similarly as in panel **c** and **d**. **g,** Both VGLL3 and TEAD are required for the ERα downregulation by VT02956. WT, *VGLL3* KO, or *TEAD1-4* qKO MCF-7 cells treated with 2 μM VT02956, 2 μM VT02484, or DMSO as control for 2 days were harvested and subjected to immunoblot with indicated antibodies. For **c**-**e**, n = 3 with mean ± SEM, Two-sided, unpaired t-test. $^{**}p<0.01$, $^{***}p<0.001$. Source data are provided as a Source Data file.

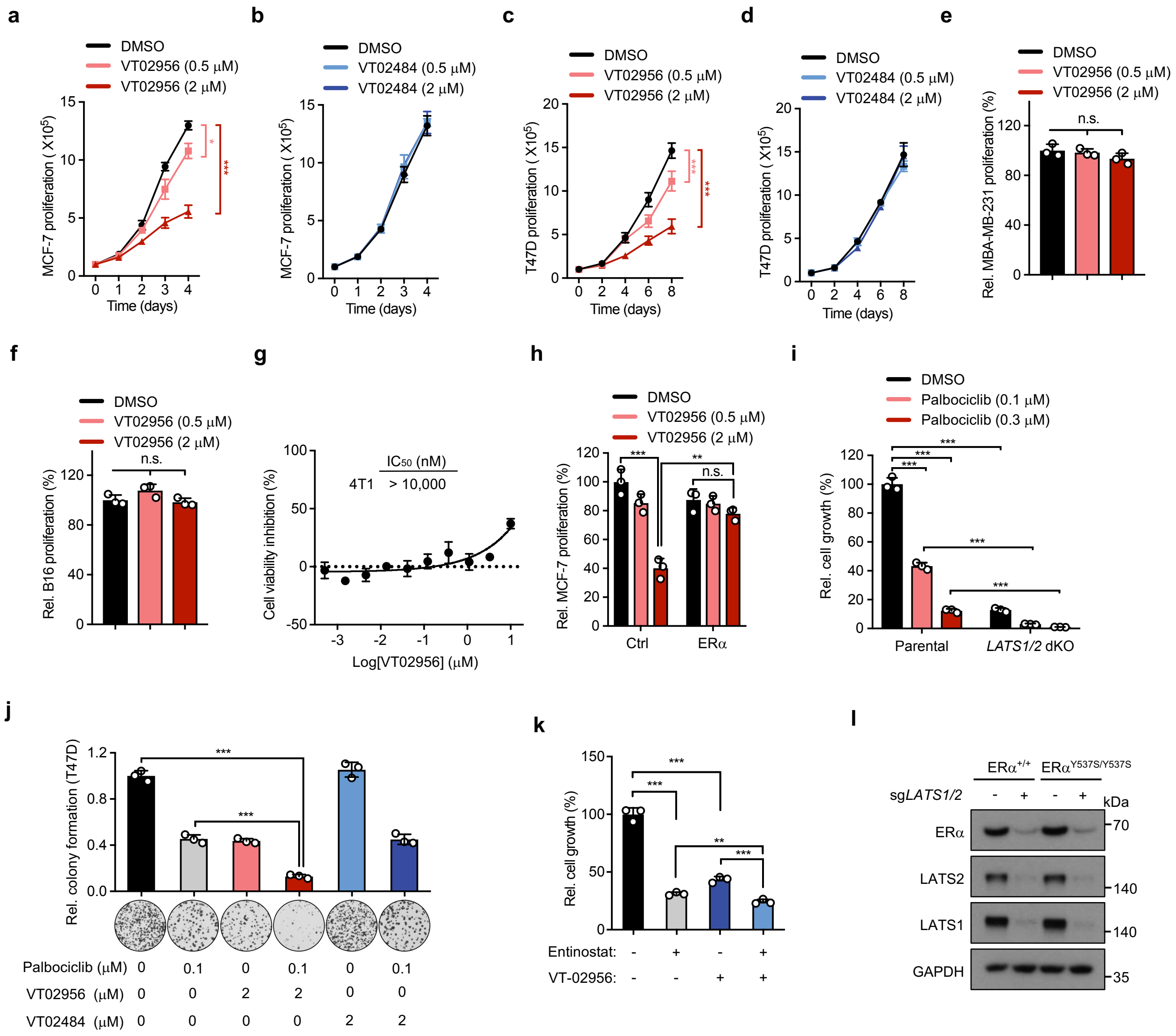
a
DMSO
VT02956 (0.5 μM)
VT02956 (2 μM)
MCF-7 proliferation (X10^5)
Time (days)
b
DMSO
VT02484 (0.5 μM)
VT02484 (2 μM)
MCF-7 proliferation (X10^5)
Time (days)
c
DMSO
VT02956 (0.5 μM)
VT02956 (2 μM)
T47D proliferation (X10^5)
Time (days)
d
DMSO
VT02484 (0.5 μM)
VT02484 (2 μM)
T47D proliferation (X10^5)
Time (days)
e
DMSO
VT02956 (0.5 μM)
VT02956 (2 μM)
n.s.
Rel. MBA-MB-231 proliferation (%)
f
DMSO
VT02956 (0.5 μM)
VT02956 (2 μM)
n.s.
Rel. B16 proliferation (%)
g
IC50 (nM)
4T1 > 10,000
Cell viability inhibition (%)
Log[VT02956] (μM)
h
DMSO
VT02956 (0.5 μM)
VT02956 (2 μM)
n.s.
Rel. MCF-7 proliferation (%)
Ctrl
ERα
i
DMSO
Palbociclib (0.1 μM)
Palbociclib (0.3 μM)
Rel. cell growth (%)
Parental
LATS1/2 dKO
j
Rel. colony formation (T47D)
Palbociclib (μM) 0 0.1 0 0.1 0 0.1
VT02956 (μM) 0 0 2 2 0 0
VT02484 (μM) 0 0 0 0 2 2
k
Rel. cell growth (%)
Entinostat: - + - +
VT-02956: - - + +
l
ERα+/+
ERαY537S/Y537S
sgLATS1/2 - + - +
kDa
ERα
LATS2
LATS1
GAPDH
70
140
140
35

Supplementary Figure 8. VT02956 suppresses ER^+ breast cancer growth.

a, **b** VT02956 but not VT02484 inhibits the growth of MCF-7 cell. Cell proliferation was assessed over a 4-day time course with the indicated concentration of compounds. **c**, **d** VT02956 but not VT02484 inhibits the T47D cell growth. Experiments were similar to those in (**a**, **b**). **e**, **f** VT02956 does not inhibit the growth of ER negative cancer cells. ER negative breast cancer MBA-MB-231 (**e**) or melanoma B16 (**f**) were treated with 0.5 μM or 2 μM VT02956 for 4 days. **g** VT02956 does not inhibit the growth of ER negative breast cancer cells 4T1. Cell viability was measured in 4T1 cells treatment with increasing concentrations of VT02956, n = 2 for each concentration dose, error bar SEM. **h** Ectopic ERα expression blocks the anti-growth effect of VT02956. MCF-7 cells ectopically expressing ERα or control were treated with 0.5 μM or 2 μM VT02956 for 4 days. Cell proliferation was measured by cell counting and normalized to DMSO treated control cells. **i** Palbociclib inhibits the growth of *LATS1/2* dKO cells. Cell growth of wild-type or *LATS1/2* deficient MCF-7 cells treated Palbociclib with indicated concentrations for 7 days. **j** VT02956 and Palbociclib show synergistic anti-cancer efficacy in ER^+ breast cancer cells. T47D cells were grown with VT02956 (2 μM), VT02484 (2 μM) or Palbociclib (0.1 μM) alone or in combination for 14 days. The panels show representative colony-formation assays of three independent experiments. **k** Effect of Entinostat and VT02956 on MCF-7 cell growth. Growth of MCF7 cell treated with DMSO control, Entinostat (1 μM), VT02956 (2 μM) or in combination for 4 days were determined by cell counting. **l** *LATS1/2* knockout decreases the expression of both wild type and hormone resistant mutant ERα. WT or ERα-Y537S knock-in MCF-7 cells with lentivirus-mediated CRISPR deletion of *LATS1/2* were subjected to immunoblot with indicated antibodies. For **a-f**, **h-I**, **k**, n = 3 with mean ± SEM. Two-way (**a**, **c**) or one-way (**e-f, h-k**) ANOVA Tukey test; *p<0.05, **p<0.01, ***p<0.001; Source data are provided as a Source Data file.

Supplementary Table 1. Kinome profiling of VT02956. Numbers indicate % enzymatic activity relative to DMSO controls

Kinases	**VT02956 (5 nM) (%)**
PRKX	3
STK38/NDR1	16
LATS1	22
PKAcg	28
STK38L/NDR2	29
LATS2	34
p70S6K/RPS6KB1	38
MSK1/RPS6KA5	49
PKN3/PRK3	64
p70S6Kb/RPS6KB2	65
PKA	68
PKN1/PRK1	69
RIPK2	75
CDK9/cyclin T2	74
PKMYT1	77
CAMK2d	77
FER	78
TYK2	78
MSK2/RPS6KA4	78
AKT1	78
BLK	78
CK2a2	78
CDK9/cyclin T1	80
WNK3	82
JAK1	82
CHK1	80
NEK11	82
PHKg2	82
BTK	83
EPHA3	84
KSR2	83
CAMKK1	83
TXK	83
AKT2	85
HGK/MAP4K4	85
RSK1	86
MUSK	85
CK2a	85
SNRK	86
IKKb/IKBKB	86
COT1/MAP3K8	86
