# Supplementary material for: Transcriptional repression of estrogen receptor alpha by YAP reveals the Hippo pathway as therapeutic target for ER+ breast cancer
Source: Nat Commun. 2022 Feb 25;13:1061. doi: 10.1038/s41467-022-28691-0 (PMC8881512; doi:10.1038/s41467-022-28691-0)
Supplement: Supplementary file 2 — Reporting Summary [file 41467_2022_28691_MOESM2_ESM.pdf]

nature portfolio

Corresponding author(s): Kun-Liang Guan

Last updated by author(s): Jan 11, 2022

Reporting Summary

Nature Portfolio wishes to improve the reproducibility of the work that we publish. This form provides structure for consistency and transparency in reporting. For further information on Nature Portfolio policies, see our Editorial Policies and the Editorial Policy Checklist.

Statistics

For all statistical analyses, confirm that the following items are present in the figure legend, table legend, main text, or Methods section.

n/a	Confirmed	
☐	☒	The exact sample size (*n*) for each experimental group/condition, given as a discrete number and unit of measurement
☐	☒	A statement on whether measurements were taken from distinct samples or whether the same sample was measured repeatedly
☐	☒	The statistical test(s) used AND whether they are one- or two-sided<br>*Only common tests should be described solely by name; describe more complex techniques in the Methods section.*
☐	☒	A description of all covariates tested
☐	☒	A description of any assumptions or corrections, such as tests of normality and adjustment for multiple comparisons
☐	☒	A full description of the statistical parameters including central tendency (e.g. means) or other basic estimates (e.g. regression coefficient) AND variation (e.g. standard deviation) or associated estimates of uncertainty (e.g. confidence intervals)
☐	☒	For null hypothesis testing, the test statistic (e.g. *F*, *t*, *r*) with confidence intervals, effect sizes, degrees of freedom and *P* value noted<br>*Give P values as exact values whenever suitable.*
☒	☐	For Bayesian analysis, information on the choice of priors and Markov chain Monte Carlo settings
☒	☐	For hierarchical and complex designs, identification of the appropriate level for tests and full reporting of outcomes
☐	☒	Estimates of effect sizes (e.g. Cohen's *d*, Pearson's *r*), indicating how they were calculated

Our web collection on statistics for biologists contains articles on many of the points above.

Software and code

Policy information about availability of computer code

Data collection	TCGA data was collected from UCSC-xena or cBioportal.
Data analysis	GraphPad (v8.0), Excel (v16.51), NIS elements imaging software (v4.11.0),ImageJ (v2.0.0), Xlfit (v5.4.0), Byonic (v4.2), fastqc (v0.11.8), trim-galore (v0.6.6), salmon (v1.1.0), DESeq2 (v1.24.0), bwa (v0.7.17), bowtie2 (v2.3.5), samtools (v1.9), bamtools (v2.5.1), MACS2.0 (v2.0.10), Easeq (v2.0), IGV (v2.9.4), MEME-Suit (5.0.1), SEACR (v1.3)<br>custom codes: Umi-4C (https://github.com/tanaylab/umi4cpackage)

For manuscripts utilizing custom algorithms or software that are central to the research but not yet described in published literature, software must be made available to editors and reviewers. We strongly encourage code deposition in a community repository (e.g. GitHub). See the Nature Portfolio guidelines for submitting code & software for further information.

Data

Policy information about availability of data

All manuscripts must include a data availability statement. This statement should provide the following information, where applicable:

- Accession codes, unique identifiers, or web links for publicly available datasets
- A description of any restrictions on data availability
- For clinical datasets or third party data, please ensure that the statement adheres to our policy

All important data generated or analyzed during this study are included in this article. The RNA sequencing data, ChIP-seq and CUT&Tag-seq data are available in GEO Database with the accession number GSE181460 https://www.ncbi.nlm.nih.gov/geo/query/acc.cgi?acc=GSE181460. Additional supplementary data are available from the corresponding author upon request.

Field-specific reporting

Please select the one below that is the best fit for your research. If you are not sure, read the appropriate sections before making your selection.

☒ Life sciences ☐ Behavioural & social sciences ☐ Ecological, evolutionary & environmental sciences

For a reference copy of the document with all sections, see nature.com/documents/nr-reporting-summary-flat.pdf

Life sciences study design

All studies must disclose on these points even when the disclosure is negative.

Sample size	No pre-determine sample-size calculations were performed. The sample size was based on the literature that tested the same cell lines or performed the similar assays. Sample size for 3 biological replicates of RNA-seq in this study were used according to common practice.
Data exclusions	No data was excluded from the experiments.
Replication	Experiments were performed 2-3 times. Similar observation was obtained for each replicate. Representative result was shown in the figures.
Randomization	No randomization was performed. Most comparisons were performed between determined status, or WT vs mutant.
Blinding	The experiments and analysis were performed in a non-blinded fashion because the same investigator was doing group allocation during data collection and analysis. Our analyses all generate objective outcomes that are not subject to observer bias.

Reporting for specific materials, systems and methods

We require information from authors about some types of materials, experimental systems and methods used in many studies. Here, indicate whether each material, system or method listed is relevant to your study. If you are not sure if a list item applies to your research, read the appropriate section before selecting a response.

Materials & experimental systems

n/a	Involved in the study	
☐	☒	Antibodies
☐	☒	Eukaryotic cell lines
☒	☐	Palaeontology and archaeology
☐	☒	Animals and other organisms
☐	☒	Human research participants
☒	☐	Clinical data
☒	☐	Dual use research of concern

Methods

n/a	Involved in the study	
☐	☒	ChIP-seq
☒	☐	Flow cytometry
☒	☐	MRI-based neuroimaging

Antibodies

Antibodies used	The antibody against Flag (HRP) (#A5892) was from Sigma. The antibody against GAPDH (#sc-25778), YAP/TAZ (#sc-101199) and ER-alpha (mouse) (#sc-542) were obtained from Santa Cruz Biotechnology. The antibody against LATS1 (#3477), LATS2 (#5888), pLATS1/2 (HM) (#8654), YAP (#14074), pYAP(S127) (#4911), NCOR2 (#62370), ER-alpha (human) (#8644) and HA-tag (#2367), Myc-tag (#2278), Flag-tag (#14793) were from Cell Signaling Technology. The antibody againt Rabbit IgG (SAB3700889) were from Sigma
Validation	Flag (HRP) (https://www.sigmaaldrich.com/GB/en/product/sigma/a8592)<br>GAPDH (https://www.scbt.com/p/gapdh-antibody-fl-335)<br>YAP/TAZ (https://www.scbt.com/p/yap-antibody-63-7)<br>ER-alpha (mouse) (https://www.scbt.com/p/eralpha-antibody-mc-20)<br>LATS1 (https://www.cellsignal.co.uk/products/primary-antibodies/lats1-c66b5-rabbit-mab/3477)<br>LATS2 (https://www.cellsignal.co.uk/products/primary-antibodies/lats2-d83d6-rabbit-mab/5888)<br>pLATS1/2 (HM) (https://www.cellsignal.co.uk/products/primary-antibodies/phospho-lats1-thr1079-d57d3-rabbit-mab/8654)<br>YAP (https://www.cellsignal.co.uk/products/primary-antibodies/yap-d8h1x-xp-rabbit-mab/14074)<br>NCOR2 (https://www.cellsignal.co.uk/products/primary-antibodies/smrt-d8d2l-rabbit-mab/62370)<br>ER-alpha (human) (https://www.cellsignal.co.uk/products/primary-antibodies/estrogen-receptor-a-d8h8-rabbit-mab/8644)<br>HA-tag (https://www.cellsignal.co.uk/products/primary-antibodies/ha-tag-6e2-mouse-mab/2367)<br>Myc-tag (https://www.cellsignal.co.uk/products/primary-antibodies/myc-tag-71d10-rabbit-mab/2278)<br>Flag-tag (https://www.cellsignal.co.uk/products/primary-antibodies/dykddddk-tag-d6w5b-rabbit-mab-binds-to-same-epitope-as-sigma-s-anti-flag-m2-antibody/14793)<br>Rabbit IgG (https://www.sigmaaldrich.com/GB/en/product/sigma/sab3700889)

Eukaryotic cell lines

Policy information about cell lines

Cell line source(s)	The MCF-7, MDA-MB-231, B16, HEK293A, T47D, 4T1 cells were from ATCC. The modified cell lines with CRISPR KO or shRNA deletion were generated in our lab.
Authentication	Cell lines were originally obtained from ATCC and therefore were not authenticated.
Mycoplasma contamination	All cells were tested for mycoplasma and they are negative of mycoplasma contamination.
Commonly misidentified lines (See ICLAC register)	MCF-7 is listed in ICLAC.

Animals and other organisms

Policy information about studies involving animals; ARRIVE guidelines recommended for reporting animal research

Laboratory animals	Lats1fl/flLats2fl/fl mice were obtained from Dr. Randy L. Johnson (MD Anderson Cancer Center, Houston, TX, USA). Female mice with age between 6-10-week-old were utilized.
Wild animals	None
Field-collected samples	None
Ethics oversight	All procedure of the mouse experiments performed were conducted with the approval of the Institutional Animal Care and Use Committee (IACUC) at the University of California, San Diego with protocol number S10259.

Note that full information on the approval of the study protocol must also be provided in the manuscript.

Human research participants

Policy information about studies involving human research participants

Population characteristics	For breast tumour orgnaoids, the samples were obtained from Biorepository and Tissue Technology Shared Resource (BTTSR), UCSD Moores Cancer Center.
Recruitment	Research samples were obtained from Biorepository and Tissue Technology Shared Resource (BTTSR), UCSD Moores Cancer Center with informed patient consent obtained pior to sample collection.
Ethics oversight	The responsible ethics committees of University of California, San Diego with IRB number 181755.

Note that full information on the approval of the study protocol must also be provided in the manuscript.

ChIP-seq

Data deposition

☒ Confirm that both raw and final processed data have been deposited in a public database such as GEO.

☒ Confirm that you have deposited or provided access to graph files (e.g. BED files) for the called peaks.

Data access links *May remain private before publication.*	All ChIP-seq data that support the findings of this paper were deposited in the GEO database with accession code GSE181460
Files in database submission	Fastq files for ChIP-seq, ATAC-seq and RNA-seq have all been deposited
Genome browser session (e.g. UCSC)	Data are available at GSE181460. Bigwig files are provided

Methodology

Replicates	Each sample were derived from the pool of 2 biological samples.
Sequencing depth	The ChIP-seq were sequenced using HiSeq-4000 to a depth of approximately 20 million reads, single-end at 75bp length.<br>The ATAC-seq were sequenced using NovaSeq-6000 to a depth of approximately 15 million reads, paired-end at 100bp length.<br>The CUT&Tag-seq were sequenced using NovaSeq-6000 to a depth of approximately 3 million reads, paired-end at 100bp length.
Antibodies	anti-ER-alpha Cell Signaling Technology 8644<br>anti-H3K4me3 Cell Signaling Technology 9727<br>anti-H3K27me3 Millipore 07-449

	anti-H3K4me1 Abcam ab8895<br>anti-H3K27ac Cell Signaling Technology 8173<br>anti-rabbit IgG Cell Signaling Technology 2729<br>anti-pPolII-S2 Abcam ab5095<br>anti-CTCF Cell Signaling Technology 3418<br>anti-H4K20me1 Abcam ab9051<br>anti-H3K9me3 Abcam ab8898<br>anti-H3K36me3 Cell Signaling Technology 4909<br>anti-HA Cell Signaling Technology 3724
Peak calling parameters	Peak calling was performed with MACS (v2.0.10) with parameter -nomodel
Data quality	FastQC (version v0.11.8) was used for sequencing quality control. Trim_galore (version 0.6.6) for quality and adapter trimming.
Software	bwa (version 0.7.17) for sequencing read mapping to hg38 genome. samtools (version 1.9) for filtering uniquely mapping reads, remove blacklist region. MACS2 (version 2.2.6) for peak calling. EaSeq for heatmap and plot profile generation.
